# Panduratin A Induces Caspase-Dependent Apoptosis and G1-Associated Cell-Cycle Arrest and Enhances TNF-α-Associated Cytotoxicity in NSCLC Cells

**DOI:** 10.3390/biom16091312

**Published:** 2026-09-10

**Authors:** Nitchakarn Phimthong, Jatuporn Polhiran, Saranyapin Potikanond, Wutigri Nimlamool, Nitwara Wikan

**Affiliations:** 1 Department of Pharmacology, Faculty of Medicine, Chiang Mai University, Chiang Mai 50200, Thailand; nitchakarn_ph@cmu.ac.th (N.P.); jatupornbam@gmail.com (J.P.); saranyapin.p@cmu.ac.th (S.P.); wutigri.nimlamool@cmu.ac.th (W.N.); 2 PhD’s Degree Program in Pharmacology, Department of Pharmacology, Faculty of Medicine, Chiang Mai University, Chiang Mai 50200, Thailand; 3 Lanna Rice Research Center, Chiang Mai University, Chiang Mai 50200, Thailand

**Keywords:** non-small-cell lung cancer (NSCLC), panduratin A, tumor necrosis factor-α (TNF-α), apoptosis, caspase-3, PARP-1, cell-cycle arrest, G1-phase, thymidine kinase, cyclin A2

## Abstract

Background: Non-small-cell lung cancer (NSCLC) is a major cause of cancer-related mortality. Current therapies are limited by drug resistance and adverse effects, highlighting the need for new strategies. Panduratin A (PA), a chalcone from *Boesenbergia rotunda*, has reported anticancer activity. This study investigated the effects of PA in NSCLC cells and whether PA enhances tumor necrosis factor alpha (TNF-α)-associated cell death. Methods: Human NSCLC cell lines A549 and H1299 were treated with PA alone or in combination with TNF-α. Cytotoxicity and apoptosis were assessed using cell-viability assays, cell-number analysis, and flow cytometry. Western blotting was used to evaluate caspase-3 activation and PARP-1 cleavage. Cell-cycle distribution was analyzed to determine the impact of PA on cell-cycle progression. Results: PA reduced cell number and induced cell death in both A549 and H1299 cells, and co-treatment with TNF-α increased apoptosis compared with single-agent treatment. PA increased the G1-phase fraction and, in a concentration-dependent manner, reduced expression of thymidine kinase and cyclin A2, consistent with a G1-associated arrest phenotype. Combination treatment also increased the sub-G1 population. Conclusions: PA promotes apoptosis in NSCLC cells and enhances TNF-α-associated apoptotic cell death in vitro, accompanied by a G1-associated cell-cycle phenotype. These findings provide an in vitro framework for studying natural compounds as modulators of TNF-α-driven apoptosis and support further mechanistic validation in more disease-relevant models.

## 1. Introduction

Lung cancer remains a significant global health burden, with non-small-cell lung cancer (NSCLC) comprising roughly 85% of all diagnoses [1,2]. NSCLC is molecularly heterogeneous, possessing genetic alterations (*EGFR*, *ALK*, *ROS1*, *KRAS*) [3,4] that have paved the way for the development of targeted drugs to improve patient outcomes. These include targeted therapies and immune-checkpoint inhibitors [4,5]. However, the efficacy of disease control may be compromised by the emergence of resistance and toxicities associated with these therapeutic agents [6]. These limitations create a major clinical challenge for NSCLC management, necessitating the development of new therapeutic strategies. Currently, research into natural compounds from medicinal herbs is expanding, revealing numerous phytochemicals with pharmacological profiles suitable for integration into novel NSCLC treatments [7,8,9,10]. In addition to single-agent activity, there is increasing interest in leveraging natural compounds as modulators of stress-response and cell-death pathways to improve tumor-cell susceptibility to cytotoxic stimuli [8,9,11].

**Figure 1:**
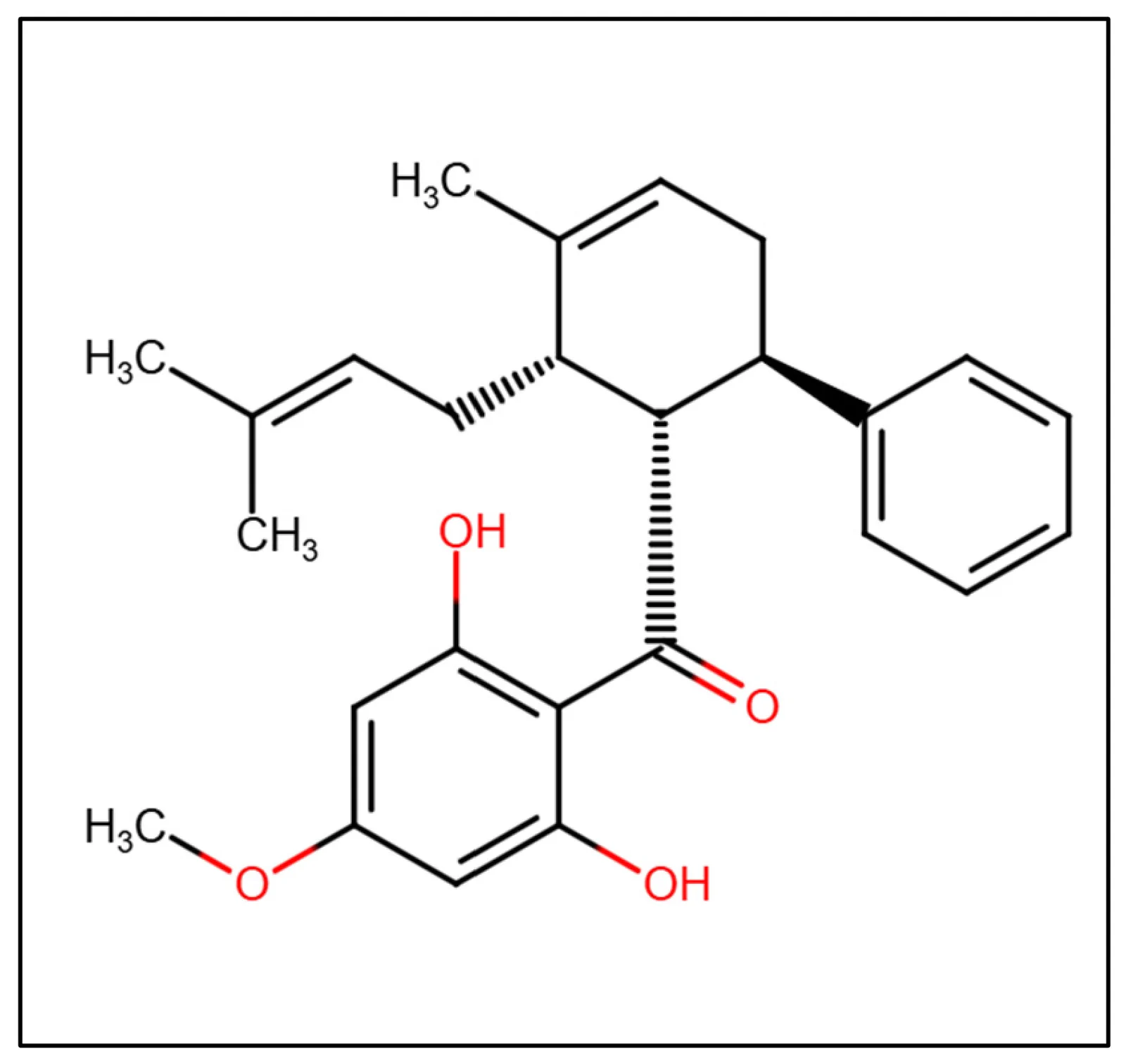
Chemical structure of panduratin A (PA).

Panduratin A (PA) is a chalcone isolated from the rhizome of *Boesenbergia rotunda* (also known as *Kaempferia pandurata* or fingerroot), a plant in the Zingiberaceae family [12]. The molecular formula of PA is C_26_H_30_O_4_ and its molecular weight is 406.51 g/mol; its chemical structure is represented in Figure 1. PA possesses a broad pharmacological profile, encompassing antioxidant, anti-inflammatory, antimicrobial, and anticancer activities [10,11,13,14,15,16]. PA exerts its effects primarily through the regulation of essential signaling cascades, including the NF-κB, MAPK, and PI3K/AKT pathways [10,11,14,16,17,18]. Among the pharmacological properties of PA, its anticancer activity is of particular interest, as this natural compound has been extensively investigated across various cancer models, including NSCLC, prostate cancer, melanoma, pancreatic cancer, lymphoma, and colorectal cancer [11,18,19,20,21,22,23,24,25]. Mechanistically, PA inhibits cancer-cell proliferation and induces apoptosis. This effect has been associated with the activation of caspase-3, alongside PARP cleavage [16,18]. PA also attenuates pro-survival inflammatory signaling by preventing IκBα degradation and the nuclear translocation of NF-κB [17], thereby downregulating the transcription of anti-apoptotic and inflammatory genes [26]. In addition, PA has been reported to exert anti-angiogenic effects through the suppression of HIF-1α/VEGF expression [17,27]. Collectively, these properties position PA as a promising candidate for deeper evaluation in NSCLC, including in combination contexts where modulation of survival signaling may alter cell-death outcomes.

Tumor necrosis factor alpha (TNF-α) is an inflammatory cytokine within the NSCLC tumor microenvironment (TME), where its elevated expression is strongly associated with poor prognosis and therapy resistance in cancer cells [28,29,30]. Upon binding to the TNF-α receptor (TNFR), TNF-α can trigger either pro-survival signaling via the IKK–NF-κB pathways or pro-apoptotic signaling through caspase activation [30,31]. In the TME context, TNF-α signaling predominantly favors the pro-survival and pro-tumorigenic axis [32,33]. NF-κB is a key transcription factor that upregulates multiple anti-apoptotic and pro-survival genes (e.g., *BCL2* family members and *XIAP*) [31,34], thereby increasing the apoptotic threshold, promoting cell survival, and limiting TNF-α–driven cytotoxicity [34]. Accumulating evidence indicates that TNF-α-mediated NF-κB activation within the tumor microenvironment triggers pro-survival signaling pathways that drive therapeutic resistance [35,36,37,38]. By impairing the efficacy of chemotherapy, targeted interventions, and immune checkpoint inhibitors, this inflammatory cascade establishes an immunosuppressive milieu that ultimately facilitates tumor progression. Therefore, we hypothesized that targeting NF-κB may shift TNF-α signaling from a pro-survival to a pro-apoptotic response by promoting activation of the caspase cascade in cancer cells. In the context of NSCLC, PA-mediated suppression of NF-κB may redirect TNF-α signaling to favor caspase activation and apoptotic cell death. However, whether PA can enhance TNF-α-induced apoptosis in NSCLC cells has not yet been reported. Therefore, we aimed to investigate the effects of PA on NSCLC cell fitness and cell-cycle progression, and to determine whether PA potentiates TNF-α-associated apoptotic cell death in NSCLC cells, using phenotypic assays and apoptosis-associated molecular markers.

## 2. Materials and Methods

### 2.1. Cell Lines and Cell Culture

Human lung cancer cell lines (NCI-H1299 (CRL-5803) and A549 (CCL-185)) were obtained from the American Type Culture Collection (ATCC) (Manassas, VA, USA). H1299 cells were cultured in ATCC-formulated RPMI-1640 medium (ATCC 30-2001) supplemented with 10% fetal bovine serum (FBS) and 1% penicillin/streptomycin (Gibco, Thermo Fisher Scientific, Waltham, MA, USA). A549 cells were cultured in ATCC-formulated F-12K medium (ATCC 30-2004) supplemented with 10% fetal bovine serum (FBS) (Gibco, Thermo Fisher Scientific, Waltham, MA, USA) and 1% penicillin/streptomycin (Gibco, Thermo Fisher Scientific, Waltham, MA, USA). Cells were cultured in a 5% CO_2_ incubator at 37 °C.

### 2.2. Chemical Structure of PA

The chemical structure of panduratin A (PA) was generated using MarvinJS (ChemAxon, Budapest, Hungary) using its canonical SMILES string retrieved from the PubChem database (CID: 6483648).

### 2.3. Cell Counting Assay

Cytotoxicity of panduratin A (PA; Biosynth^®^, Staad, Switzerland; HPLC purity 99%; Cat. No. XP180835; Lot No. 0000047260) was evaluated using a Hoechst staining method. H1299 and A549 cells (1 × 10^4^ cells/well) were seeded into 96-well plates in complete culture medium and incubated overnight. Cells were then treated with PA (0–40 µM; serial dilution), PA (0–40 µM) combined with TNF-α (20 ng/mL), or vehicle control (DMSO; Merck KGaA, Darmstadt, Germany). TNF-α (20 ng/mL) alone was also included as a control. The rationale for using TNF-α at a fixed concentration of 20 ng/mL was based on prior studies in NSCLC models showing that this dose can induce measurable TNF-α-associated apoptotic/cytotoxic responses in A549 cells over 24–48 h and has also been used in combination with bioactive compounds to assess modulation of TNF-α responses in lung-cancer cells [39,40].

Cells were treated for 24 or 48 h. For Hoechst staining, 50 µL of Hoechst 33342 solution (Merck KGaA) was added to each well, and plates were incubated at room temperature protected from light for at least 5 min. Stained cells were visualized using a BioTek Lionheart FX system (Agilent Technologies, Santa Clara, CA, USA), and cell counting was performed using BioTek Gen5 software (version 3.17, Agilent Technologies, Santa Clara, CA, USA). Cell numbers were reported relative to the vehicle control.

### 2.4. Western Blot Analysis

H1299 and A549 cells ($5\times 10^{4}\;cells/well$) were seeded into 24-well plates and incubated overnight under standard culture conditions. For detection of protein markers, the cells were treated with PA (0, 5, and 10 µM) in combination with TNF-α (20 ng/mL) for 24 or 48 h. For these experiments, PA alone and TNF-α alone conditions were also included. After the treatment was completed, cell lysates were collected using a Laemmli sample buffer and boiled at 100 °C for 5 min prior to being stored at −20 °C until the western blot was performed. Proteins were separated by sodium dodecyl sulfate–polyacrylamide gel electrophoresis (SDS-PAGE). After that, proteins were transferred onto a polyvinylidene difluoride (PVDF) membrane, and the membrane was blocked with 5% bovine serum albumin (BSA) for 1 h prior to incubation with primary antibodies at 4 °C overnight with shaking. Consequently, the membrane was washed using 0.05% Tris-buffered saline with Tween-20 (TBS-T) and incubated with the secondary antibodies for 2 h at room temperature with orbital shaking, followed by washing with TBS-T three times. The immunoreactive bands were detected using Odyssey^®^ CLx system (LICORbio, Lincoln, NE, USA), and the quantification of band intensity was performed using ImageJ software (version 1.54g, National Institutes of Health, Bethesda, MD, USA). Primary antibodies against targeted proteins purchased from Cell Signaling Technology (Danvers, MA, USA) were caspase-3, (Catalog No. 9662), PARP-1 (Catalog No. 9542), cyclin A2 (Catalog No. 67955), and Thymidine kinase (Catalog No. 28755). An antibody against β-actin (Catalog No. MA1115) was purchased from Boster Biological Technology (Pleasanton, CA, USA). Secondary antibodies purchased from LI–COR Biosciences (Lincoln, NE, USA) were IRDye^®^ 800CW goat anti-mouse (Catalog No. 926-32210) and IRDye^®^ 680RD goat anti-rabbit (Catalog No. 926-68071). PARP-1 and caspase-3 cleavage products were evaluated as apoptosis-associated molecular markers.

### 2.5. Immunofluorescence Study

H1299 and A549 cells ($5\;\times {\;10}^{4}\;\mathrm{and}\;7\;\times \;10^{4}\;\mathrm{cells}/\mathrm{well},\;\mathrm{respectively}$) were seeded onto round coverslips in 24-well plates and allowed to attach to the coverslip surface overnight. Cells were treated with PA (10 µM), TNF-α (20 ng/mL), or the combination of PA and TNF-α for 24 h. Cells were then fixed with 4% formaldehyde, and permeabilization was performed using 0.3% Triton X-100 (Amresco LLC, Solon, OH, USA) prior to incubation with primary antibodies against cyclin A2 (Cell Signaling Technology, Catalog No. 67955) and thymidine kinase (TK) (Cell Signaling Technology, Catalog No. 28755) at 4 °C overnight. After primary antibody incubation, coverslips were washed three times with phosphate-buffered saline (PBS) and incubated with a secondary antibody (Alexa Fluor 488 goat anti-rabbit IgG [H + L]; Thermo Fisher Scientific, Waltham, MA, USA, Catalog No. A11008) mixed with DAPI (4′,6-diamidino-2-phenylindole; Cell Signaling Technology) and DyLight 594 Phalloidin (Cell Signaling Technology). Image visualization was performed using a Leica DMi8 Thunder Imager 3D microscope (Leica Microsystems, Wetzlar, Germany) equipped with LAS X image-processing software (version 3.8.1; Leica Microsystems Ltd.) using a 100× oil-immersion objective. Images from all treatment groups within each experiment were acquired using identical imaging settings, including the objective lens, exposure time, illumination intensity, and detector gain. No treatment-specific adjustment of image-acquisition parameters was applied. Where brightness and contrast adjustments were used for figure preparation, these adjustments were applied uniformly across all treatment groups within the same staining experiment, without selective enhancement of individual images.

For immunofluorescence quantification, fluorescence intensity was measured on a per-cell basis using ImageJ software (version 1.54g, National Institutes of Health, Bethesda, MD, USA). For cyclin A2 and TK immunofluorescence, cells were manually outlined to define regions of interest (ROIs). Background fluorescence was subtracted before analysis. For TK, mean fluorescence intensity was quantified within whole-cell ROIs defined by phalloidin staining, whereas cyclin A2 mean fluorescence intensity was quantified within DAPI-defined nuclear ROIs. At least 50 cells in total, sampled across five randomly selected fields, were analyzed for each condition in each biological replicate. The mean value across quantified cells from each biological replicate was used for statistical analysis. Fluorescence-intensity values were expressed relative to the untreated control (UT set to 1.0).

### 2.6. Flow Cytometry

Cells ($2\;\times \;10^{5}\;\mathrm{cells}/\mathrm{well}$) were seeded in 24-well plates and incubated overnight. PA and PA combined with TNF-α were prepared by diluting them in culture medium supplemented with 10% FBS and antibiotics. TNF-α alone was also included as a control. The cells were treated for 24 h and/or 48 h, depending on the assay endpoint. For apoptosis analysis, 24 h was included to capture early changes in apoptotic dynamics, whereas 48 h was used to quantify robust apoptotic accumulation. For cell-cycle analysis, cells were analyzed at 24 h to capture early cell-cycle redistribution before extensive secondary apoptosis (sub-G1 accumulation) at later time points could confound PI DNA-content profiling. Cells were harvested by trypsinization for 5 min and centrifugation at 4 °C, at 350 g for 5 min. Then, the supernatant was discarded. In order to observe apoptotic cell death, binding buffer was added to the samples, followed by adding Annexin-V-FITC (ImmunoTools^®^, Friesoythe, Germany) and propidium iodide (PI) (Sigma-Aldrich, St. Louis, MO, USA) at a ratio of 5:1. Apoptotic populations were defined as follows: LR (Annexin V+/PI−) = early apoptosis and UR (Annexin V+/PI+) = late apoptosis; total apoptosis was calculated as LR + UR. For the purpose of cell-cycle analysis, the cells were fixed and permeabilized with cold 70% ethanol at −20 °C overnight and washed with PBS prior to flow cytometry. For cell-cycle analysis, cells were stained with a PI-based DNA content-staining solution with RNase treatment prior to acquisition to determine the distribution of sub-G1, G0/G1, S, and G2/M populations. Flow cytometry was performed using a Beckman Coulter DxFLEX flow cytometer (Beckman Coulter, Inc., Miami, FL, USA). The percentage of apoptotic cells and the ratio of cell populations were analyzed using CytExpert for DxFLEX software (version 2.0.0.283, Beckman Coulter, Inc., Miami, FL, USA).

### 2.7. Bliss Independence Model Calculation

The interaction between PA and TNF-α was evaluated using the Bliss independence model. Bliss independence analysis was performed to evaluate the interaction between increasing concentrations of PA and a fixed concentration of TNF-α (20 ng/mL). After obtaining cell numbers following treatment with PA, TNF-α, and PA + TNF-α, we calculated the fractional inhibition of PA (EP), TNF-α (ET), and PA + TNF-α (EPT). After that, the Bliss expected value was calculated as Bliss expected = E_P + E_T-(E_P × E_T). Consequently, the Bliss score was calculated as Bliss score = E_PT-E_exp. The positive Bliss score indicated synergy, values near 0 indicated additivity, and negative values were antagonism.

### 2.8. In Silico ADME and Drug-Likeness Analysis

To predict the pharmacokinetic profile, ADME (absorption, distribution, metabolism, and excretion) properties, and drug-likeness of panduratin A (PA), an in silico profiling, was conducted using the SwissADME web server http://www.swissadme.ch/ (accessed on 13 August 2026). The chemical structure and canonical SMILES string of PA (PubChem CID: 6483648) were retrieved from the PubChem database. The SMILES notation was then submitted to the SwissADME platform to calculate key physicochemical descriptors, including molecular weight (MW), topological polar surface area (TPSA), rotatable bonds, hydrogen bond donors/acceptors, and lipophilicity (consensus Log $P_{o/w}$). Pharmacokinetic parameters comprising gastrointestinal (GI) absorption, blood–brain barrier (BBB) permeability, P-glycoprotein (P-gp) substrate interaction, and cytochrome P450 (CYP1A2, CYP2C19, CYP2C9, CYP2D6, and CYP3A4) enzyme inhibition profiles were computationally predicted. Furthermore, the drug-likeness of PA was evaluated against established medicinal chemistry filters, including Lipinski’s Rule of Five, Veber, Egan, and Muegge criteria, alongside the Abbott bioavailability score and Pan-Assay Interference Compounds (PAINS) structural alerts.

### 2.9. Statistical Analysis

Statistical analyses were performed using GraphPad Prism (version 10.0.0). Data are presented as mean ± SEM from *n* = 3 independent biological replicates per condition (exact *n* values are indicated in the figure legends). Statistical significance was defined as α = 0.05 (95% confidence). Data were assessed for normality (Gaussian distribution) as appropriate prior to parametric testing. For datasets involving two independent factors (e.g., PA concentration/dose × TNF-α treatment (±TNF-α), or treatment condition × assay quadrant), data were analyzed using ordinary two-way ANOVA (α = 0.05). When significant interaction and/or main effects were observed, post hoc multiple-comparisons testing was performed as specified below and in the corresponding figure legends. For cell-count/viability experiments analyzed in a two-factor design, Tukey’s multiple-comparisons test was used to compare treatments within each PA concentration. For apoptosis four-quadrant analyses, Tukey’s multiple-comparisons test was used to evaluate simple effects within each quadrant (i.e., comparisons of conditions within each quadrant). For cell-cycle distribution, analyses were performed separately for each cell line and for each cell-cycle phase (G1, S, G2/M, and sub-G1). When cell-cycle experiments involved two factors (PA concentration × TNF-α treatment), each phase was analyzed using ordinary two-way ANOVA followed by Šídák’s multiple-comparisons test for planned comparisons versus (i) untreated control and (ii) TNF-α alone, as specified in the figure legends. In experiments comparing four treatment groups (untreated control, TNF-α, PA, and PA + TNF-α) within a single factor, each phase was analyzed using ordinary one-way ANOVA followed by Tukey’s multiple-comparisons test. For protein-expression measurements analyzed in a two-factor design (PA concentration × TNF-α treatment), analyses were performed separately for each cell line using ordinary two-way ANOVA followed by Šídák’s multiple-comparisons test to compare (i) each treatment versus untreated control and (ii) PA + TNF-α versus TNF-α alone at matched PA concentrations. This Šídák approach was used in place of uncorrected Fisher’s LSD to control for multiple comparisons. For immunofluorescence quantification, five fields were quantified per biological replicate and averaged to yield a single value per replicate per condition prior to statistical testing.

## 3. Results

### 3.1. Panduratin A Enhances the TNF-α-Mediated Reduction in NSCLC Cell Number

**Figure 2:**
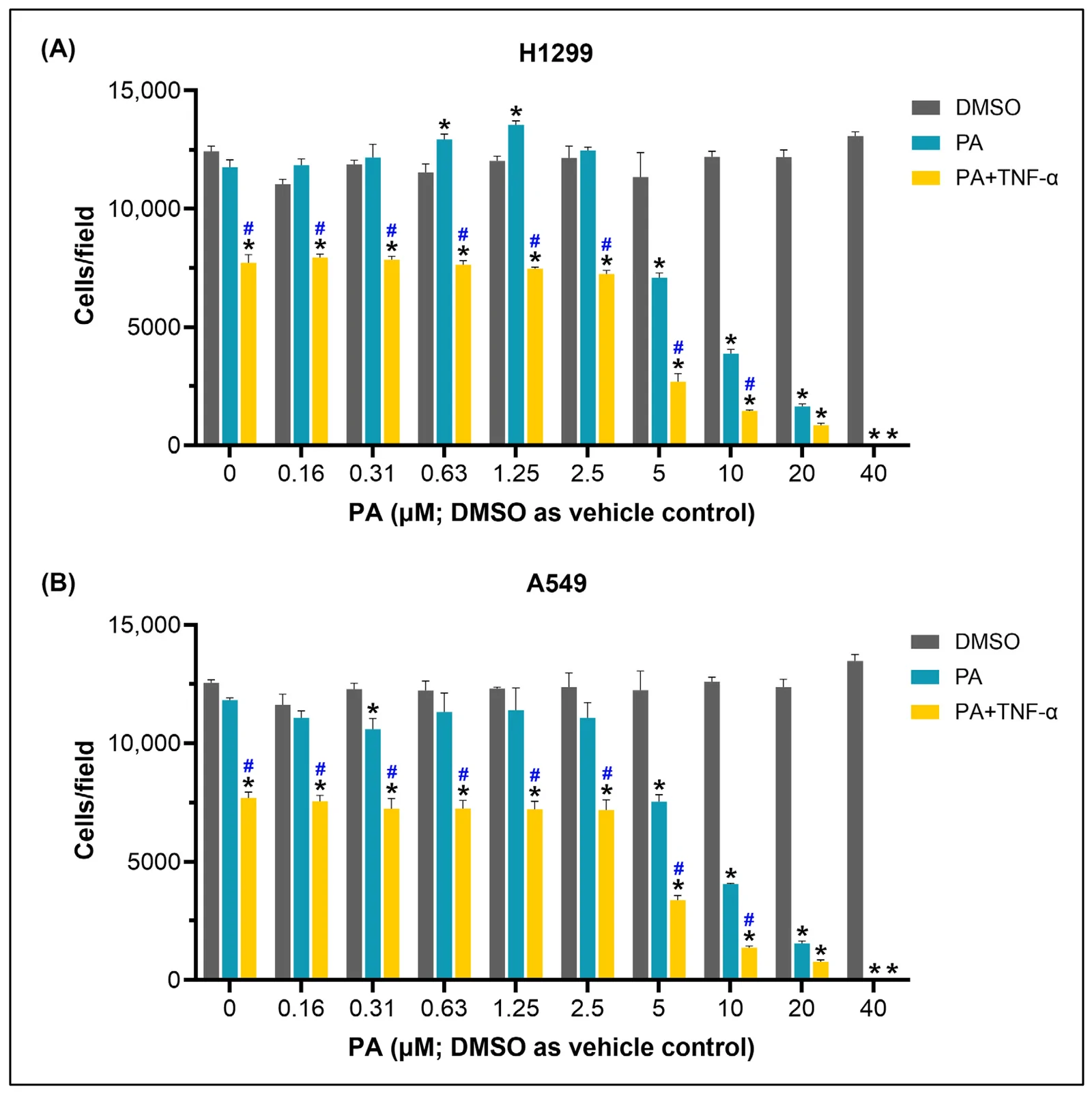
PA reduces cells/field in a dose-dependent manner, and co-treatment with TNF-α further decreases cells/field in H1299 (**A**) and A549 (**B**) cells. Cells were treated for 48 h with DMSO (PA vehicle control), PA (0–40 µM), or PA + TNF-α (20 ng/mL; TNF-α held constant in the combination condition). At 0 µM PA, the PA-alone condition corresponds to the DMSO PA vehicle control (no PA and no TNF-α), whereas the PA + TNF-α condition corresponds to TNF-α treatment alone (20 ng/mL, without PA). Therefore, these conditions represent distinct treatments and are not expected to have identical cell-number values. Nuclei were stained with Hoechst 33342 and imaged using a 4× objective, and cell number was quantified by automated nuclear counting (cells/field). Data are shown as mean ± SEM from *n* = 3 independent biological replicates per condition. For each cell line, statistical analysis was performed using ordinary two-way ANOVA (α = 0.05) with factors PA concentration (10 levels) and treatment (DMSO, PA, PA + TNF-α), followed by Tukey’s multiple-comparisons test to compare treatments within each PA concentration. Two-way ANOVA detected significant main effects of dose and treatment and a significant interaction in both cell lines (H1299: interaction F(18, 60) = 84.51, *p* < 0.0001; dose F(9, 60) = 239.7, *p* < 0.0001; treatment F(2, 60) = 1279, *p* < 0.0001; A549: interaction F(18, 60) = 39.55, *p* < 0.0001; dose F(9, 60) = 114.4, *p* < 0.0001; treatment F(2, 60) = 877.4, *p* < 0.0001). For each PA concentration, * *p* < 0.05 versus DMSO and # *p* < 0.05 versus PA alone (Tukey-adjusted); unmarked comparisons were not significant. “Dose” indicates the PA concentration (10 levels), “treatment” indicates the treatment condition (DMSO, PA, or PA + TNF-α), and “interaction” (dose × treatment) tests whether the effect of PA concentration differs among treatment conditions. F(df_1_, df_2_) denotes the ANOVA F-statistic, calculated as the ratio of the mean square for the effect to the residual mean square, with the corresponding numerator and denominator degrees of freedom.

To determine the effects of PA, TNF-α, and their combination on NSCLC cell number, H1299 and A549 cells were quantified by Hoechst-based nuclear staining followed by automated counting (cells/field). Cells were treated for 48 h with PA (0–40 µM) either alone or in combination with TNF-α (20 ng/mL; fixed for the combination condition). For clarity, the 0 µM PA condition corresponds to the DMSO vehicle control (no PA). Nuclei were stained with Hoechst 33342 and imaged using a 4× objective. One field per well was acquired from 96-well plates (three replicate wells per condition), and automated cell counts were obtained using BioTek Gen5 software. As shown in Figure 2, PA reduced cells/field in a dose-dependent manner in both H1299 and A549 cells, and co-treatment with TNF-α further decreased cells/field relative to PA alone at matched PA concentrations. Consistent with this, ordinary two-way ANOVA (dose × treatment) revealed significant main effects of dose and treatment, as well as a significant interaction, in both cell lines (H1299: interaction F(18, 60) = 84.51, *p* < 0.0001; dose F(9, 60) = 239.7, *p* < 0.0001; treatment F(2, 60) = 1279, *p* < 0.0001; A549: interaction F(18, 60) = 39.55, *p* < 0.0001; dose F(9, 60) = 114.4, *p* < 0.0001; treatment F(2, 60) = 877.4, *p* < 0.0001). Tukey’s multiple-comparisons test identified PA concentrations at which the combination treatment differed significantly from the DMSO control (*) and from PA alone (#), whereas unmarked comparisons were not significant (ns). Nonlinear regression of PA dose–response curves (variable-slope 4PL) yielded IC_50_ values of 5.104 µM (95% CI 4.382–6.026 µM) for H1299 and 6.073 µM (95% CI 4.834–8.614 µM) for A549 under PA-alone conditions. In the presence of TNF-α, the apparent potency of PA increased, with IC_50_ values of 3.907 µM for H1299 (95% CI lower bound 3.587 µM; upper bound not determined by profile likelihood) and 4.316 µM for A549 (95% CI lower bound 3.926 µM; upper bound not determined). Together, these results indicate that TNF-α enhances the reduction in cell number produced by PA, producing a greater decrease in cells/field than PA alone at matched PA concentrations.

To investigate whether the combination of PA and TNF-α exerts a synergistic effect on cell-number reduction, we employed the Bliss independence model [41] as a quantitative measure of drug interaction. Bliss independence analysis was performed to evaluate the interaction between increasing concentrations of PA and a fixed concentration of TNF-α. Under Bliss independence, the expected combined fractional inhibition is calculated from the single-agent inhibitions, and synergy is indicated when the observed combined inhibition exceeds the Bliss-expected inhibition (Bliss score = $E_{\mathrm{obs}}-E_{\mathrm{Bliss}}$ > 0). As shown in Table 1, the PA + TNF-α combination produced positive Bliss scores at PA concentrations from 0.31 to 20 µM in both H1299 and A549 cells, supporting a synergistic interaction over this range. In contrast, Bliss scores were slightly negative at 0.16 µM and approached 0 at the highest PA concentration (40 µM), consistent with minimal single-agent activity at very low doses and a ceiling effect at very high doses. Notably, the strongest synergy was observed at 5 µM PA combined with TNF-α (20 ng/mL) (maximum Bliss scores: 0.173 in H1299 and 0.177 in A549), and synergy remained evident at 10 µM PA (Bliss scores: 0.089 in H1299 and 0.091 in A549). Based on this dose-response and interaction profile, subsequent experiments were focused on the effective synergy window of 5–10 µM PA, and an intermediate concentration (7.5 µM) was included to better resolve dose-dependent effects within this range.

### 3.2. PA Potentiates TNF-α-Mediated Apoptotic Cell Death in NSCLC Cells

**Figure 3:**
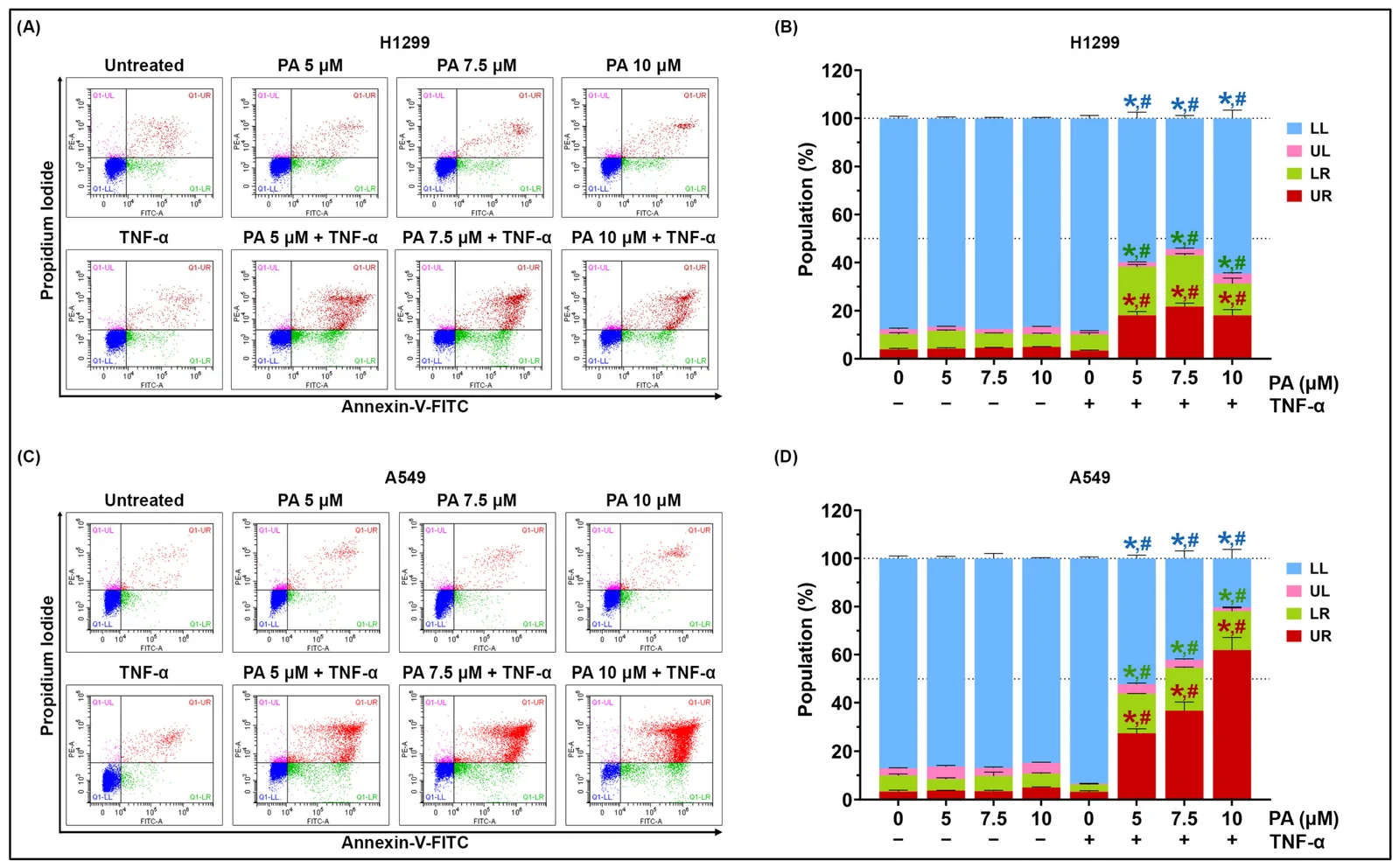
PA enhances TNF-α-induced apoptotic cell death in H1299 and A549 cells after 24 h. Representative flow cytometry dot plots of Annexin V-FITC/propidium iodide (PI) staining are shown for H1299 (**A**) and A549 (**C**). Quantification of cell-death populations is shown for H1299 (**B**) and A549 (**D**). The lower-left (LL) quadrant, Annexin V−/PI− (blue), indicates viable cells; the lower-right (LR) quadrant, Annexin V+/PI− (green), indicates early apoptosis; Annexin V−/PI+ (pink) indicates primary necrosis (UL quadrant), and Annexin V+/PI+ (red) indicates late apoptosis (UR quadrant). Cells were treated for 24 h with PA (0, 5, 7.5, or 10 µM), TNF-α (20 ng/mL), or PA plus TNF-α. Data are presented as mean ± SEM from *n* = 3 independent biological replicates per condition. Statistical analyses were performed separately for each cell line and separately for each cell-death population (viable, early apoptosis, primary necrosis, late apoptosis) using ordinary two-way ANOVA (α = 0.05) with factors PA dose and TNF-α (− or +; 20 ng/mL), followed by Tukey’s multiple-comparisons test comparing conditions within each PA dose. In the graphs, * indicates *p* < 0.05 versus untreated (PA 0 µM, TNF-α−), and # indicates *p* < 0.05 versus TNF-α alone at the matched PA concentration.

**Figure 4:**
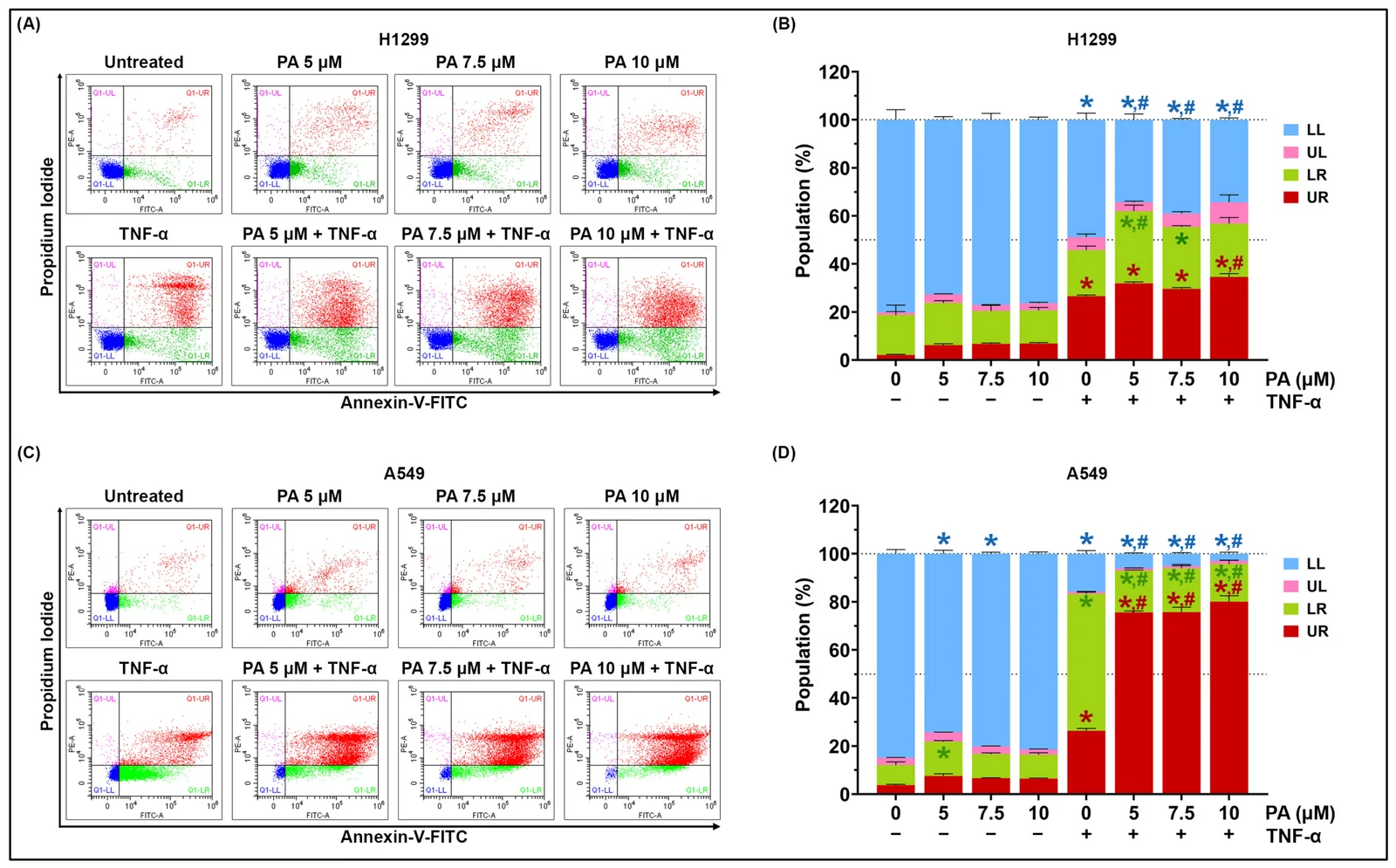
PA enhances TNF-α-induced apoptotic cell death in H1299 and A549 cells after 48 h. Representative flow cytometry dot plots of Annexin V-FITC/propidium iodide (PI) staining are shown for H1299 (**A**) and A549 (**C**). Quantification of cell-death populations is shown for H1299 (**B**) and A549 (**D**). The lower-left (LL) quadrant, Annexin V−/PI− (blue), indicates viable cells; the lower-right (LR) quadrant, Annexin V+/PI− (green), indicates early apoptosis; Annexin V−/PI+ (pink) indicates primary necrosis (UL quadrant), and Annexin V+/PI+ (red) indicates late apoptosis (UR quadrant). Cells were treated for 48 h with PA (0, 5, 7.5, or 10 µM), TNF-α (20 ng/mL), or PA plus TNF-α. Data are presented as mean ± SEM from *n* = 3 independent biological replicates per condition. Statistical analyses were performed separately for each cell line and separately for each cell-death population (viable, early apoptosis, primary necrosis, late apoptosis) using ordinary two-way ANOVA (α = 0.05) with factors PA dose and TNF-α (− or +; 20 ng/mL), followed by Tukey’s multiple-comparisons test comparing conditions within each PA dose. In the graphs, * indicates *p* < 0.05 versus untreated (PA 0 µM, TNF-α−), and # indicates *p* < 0.05 versus TNF-α alone at the matched PA concentration.

We hypothesized that the reduction in cancer-cell number following treatment may result from the induction of cell death. To test this, we employed flow cytometric analysis to determine whether apoptosis is triggered by PA, TNF-α, or their combination. Apoptosis was assessed by Annexin V-FITC/propidium iodide (PI) staining, and total apoptosis was defined as the sum of the early- and late-apoptotic populations (LR + UR). Our results demonstrated that after 24 h of treatment, untreated cells presented approximately 10% total apoptosis (LR + UR) in both H1299 and A549 (Figure 3A–D). Consistent with this low basal level, treatment with PA alone (all concentrations) and TNF-α alone at 24 h showed no significant difference when compared to untreated cells. (Figure 3A–D). Interestingly, combination treatment of PA with TNF-α increased total apoptosis significantly when compared to either untreated or TNF-α alone by approximately 20% in H1299 cells treated with PA at all concentrations (Figure 3A,B) and 20%, 35%, and 60% in A549 cells treated with PA 5, 7.5, and 10 µM respectively (Figure 3C,D). Moreover, we further investigated treatment exposure for 48 h. Although the lowest PA concentration tested here is close to the 48-h IC50 estimated from the cell-counting assay, Annexin V/PI staining provides a mechanistic snapshot of apoptotic membrane changes at a single time point and therefore is not expected to scale directly with the cell-counting endpoint, which reflects the net effect of treatment on cell accumulation over time, particularly if reduced cell number is driven largely by growth inhibition and/or loss of detached cells prior to analysis. The results demonstrated that after 48 h, untreated cells exhibited low basal apoptosis, accounting for approximately 20% and 10% of total cells in H1299 (Figure 4A,B) and A549 (Figure 4C,D) cells, respectively. Treatment with PA alone (5, 7.5, and 10 µM) did not significantly increase the apoptotic percentage in either H1299 or A549 cells, with the exception of 5 µM PA in A549 cells, which increased total apoptosis (Figure 4D). Importantly, TNF-α single-agent treatment produced a markedly different apoptotic response in the two cell lines at 48 h, with TNF-α (20 ng/mL) significantly increasing total apoptosis in H1299 cells to approximately 45% (20% early and 25% late apoptosis; Figure 4A,B), while increasing total apoptosis in A549 cells to approximately 80% (55% early and 25% late apoptosis; Figure 4C,D). This indicates a higher baseline sensitivity of A549 cells to TNF-α-associated apoptosis compared with H1299 cells under identical exposure conditions.

Furthermore, co-treatment with 20 ng/mL TNF-α and PA (5, 7.5, and 10 µM) significantly elevated total apoptosis in H1299 cells to approximately 60% with 5 µM PA (30% early apoptosis and 30% late apoptosis), ~50% with 7.5 µM PA (25% early and 25% late apoptosis), and ~50% with 10 µM PA (20% early and 30% late apoptosis), respectively (Figure 4A,B). In A549 cells, co-treatment with TNF-α and PA (5, 7.5, and 10 µM) elevated total apoptosis to roughly 95% and induced a marked shift from early to late apoptosis (15% early and 80% late; Figure 4C,D), indicating that PA potentiates TNF-α-mediated apoptotic cell death in A549 cells.

### 3.3. PA Combined with TNF-α Activates Caspase-3 and Promotes PARP-1 Cleavage in H1299 and A549 Cells

**Figure 5:**
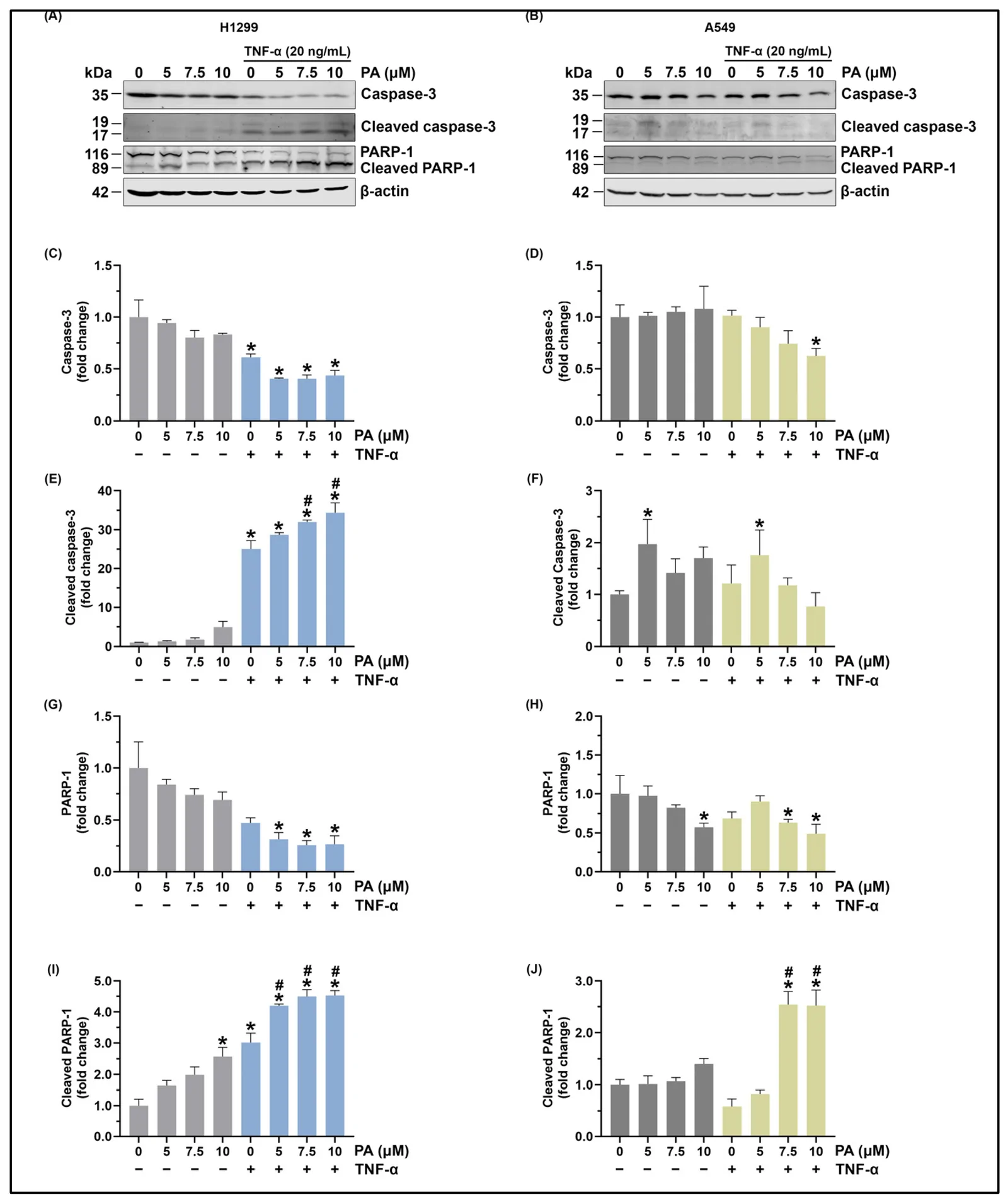
Effects of PA and PA + TNF-α on apoptotic markers in H1299 (**left** panels) and A549 (**right** panels) cells after 48 h treatment. Representative immunoreactive bands from Western blot analysis of apoptosis-related proteins in H1299 (**A**) and A549 (**B**) cells. Beta (β)-actin was used as a loading control for normalization. Densitometric quantification is shown for full-length caspase-3 in H1299 (**C**) and A549 (**D**), cleaved caspase-3 in H1299 (**E**) and A549 (**F**), full-length PARP-1 in H1299 (**G**) and A549 (**H**), and cleaved PARP-1 in H1299 (**I**) and A549 (**J**). Data are presented as mean ± SEM from *n* = 3 independent biological replicates per condition. Statistical analyses were performed separately for each cell line and each protein target using ordinary two-way ANOVA (α = 0.05) with factors PA dose (0, 5, 7.5, and 10 µM) and TNF-α (− or +; 20 ng/mL). When a significant interaction and/or main effects were detected, post hoc multiple comparisons were performed using Šídák’s test to compare conditions within each PA dose, including planned comparisons of TNF-α-treated groups versus their corresponding TNF-α-free controls at matched PA concentrations and PA + TNF-α versus TNF-α alone. In the graphs, * indicates *p* < 0.05 versus untreated control and # indicates *p* < 0.05 versus TNF-α alone (multiplicity-adjusted); unmarked comparisons are not significant (ns). The original Western blot images can be found in the Supplementary Materials.

To validate the flow cytometry results, we assessed key intracellular markers of apoptosis. Specifically, levels of caspase-3 (full-length and cleaved) and PARP-1 (full-length and cleaved) in H1299 and A549 cells were analyzed by Western blot (Figure 5A,B) after 48 h of treatment. In H1299 cells, the band intensity of full-length caspase-3 was examined. A significant decrease in full-length caspase-3 was observed when H1299 cells were treated with TNF-α in combination with PA (5, 7.5, and 10 µM), accompanied by a parallel increase in its cleaved form. Interestingly, co-treatment with PA (7.5 and 10 µM) plus TNF-α significantly increased cleaved caspase-3 compared with TNF-α alone, indicating that PA potentiates TNF-α-induced caspase-3 activation (Figure 5C,E). The pro-apoptotic effects of PA and TNF-α in H1299 cells were further substantiated by the processing of PARP-1. Specifically, PA treatment alone induced a decrease in full-length PARP-1 (Figure 5A,G) with a concomitant, concentration-dependent increase in cleaved PARP-1 (Figure 5A,I). The combination of PA and TNF-α significantly enhanced the cleavage of full-length PARP-1 and the subsequent generation of its cleaved fragment (Figure 5A,G,I), compared with the corresponding PA-only condition and/or TNF-α alone, consistent with the planned within-dose comparisons described in the Figure 5 legend. In A549 cells, caspase-3 and PARP-1 showed reductions in their full-length forms (Figure 5B,D,H). However, the cleaved caspase-3 band was weakly detectable in the representative A549 blot despite the strong apoptotic response detected by Annexin V/PI at 48 h, which may reflect limited recovery of protein from the remaining adherent cell population at this late time point under conditions of extensive cell loss. Consistent with the representative blot, PARP-1 cleavage in A549 cells was more pronounced with PA + TNF-α than with TNF-α alone, and the effect of TNF-α alone was therefore interpreted cautiously (Figure 5B,F,J). Overall, these biochemical data support the flow cytometry findings that PA enhances TNF-α-mediated apoptotic signaling.

### 3.4. PA Induces G1-Phase Arrest and Enhances TNF-α-Associated Sub-G1 Accumulation with Downregulation of G1/S Markers in NSCLC Cells

**Figure 6:**
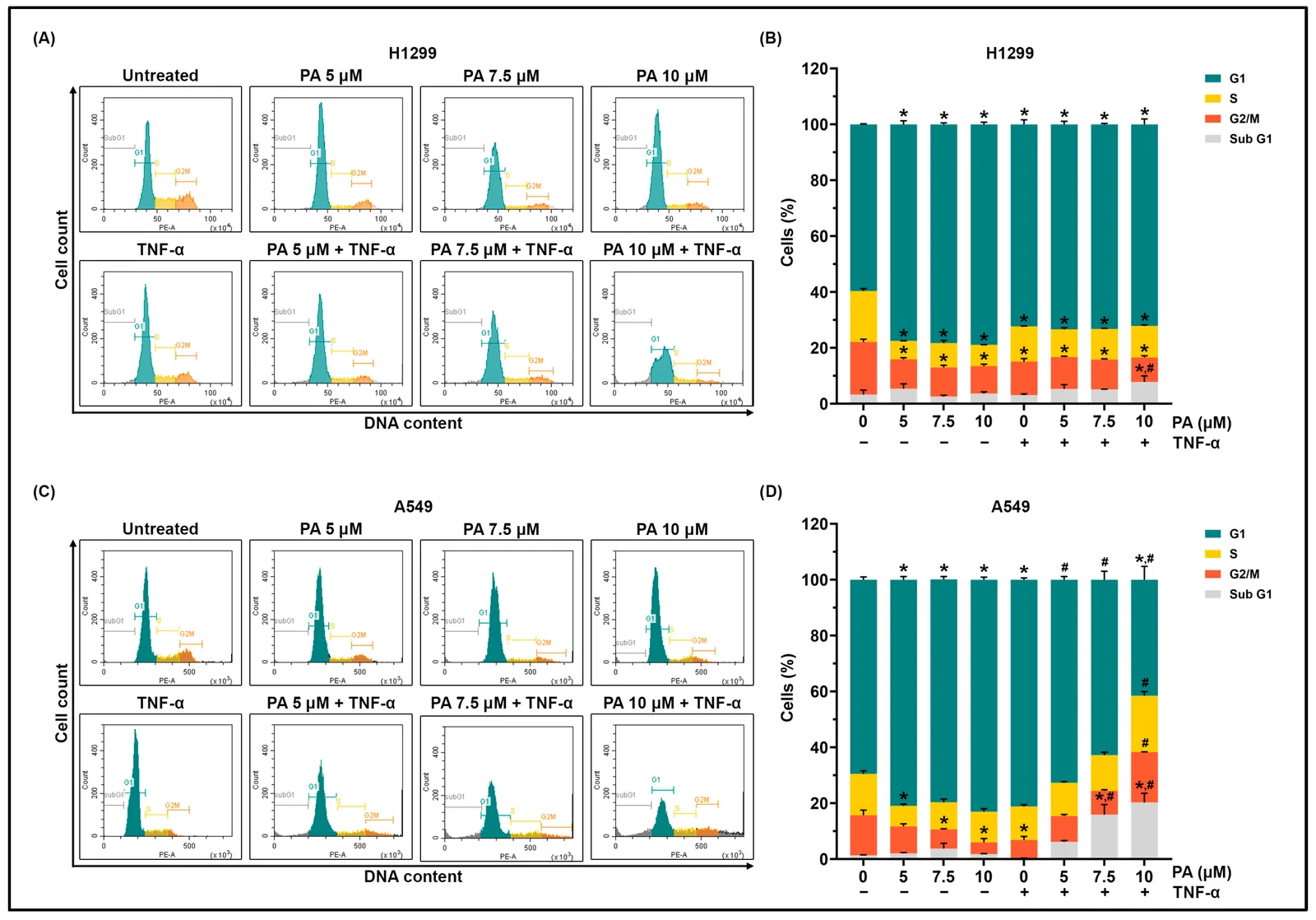
PA and PA + TNF-α modulate cell-cycle distribution in H1299 and A549 cells after 24 h of treatment. Cell-cycle profiles were determined by flow cytometry based on DNA content. Representative DNA-content histograms are shown for H1299 (**A**; **upper** panel) and A549 (**C**; **lower** panel) cells after the indicated treatments, and the corresponding quantification of the percentage of cells in G1, S, G2/M, and sub-G1 is presented for H1299 (**B**) and A549 (**D**). Cells were treated with PA (0, 5, 7.5, or 10 µM) in the absence or presence of TNF-α (20 ng/mL) for 24 h. Data are presented as mean ± SEM from *n* = 3 independent biological replicates per condition. Statistical analyses were performed separately for each cell line and for each cell-cycle phase (G1, S, G2/M, and sub-G1) using ordinary two-way ANOVA (factors: PA dose and TNF-α treatment), followed by Šídák’s multiple-comparisons test. In the graphs, * indicates *p* < 0.05 versus untreated control (PA 0 µM, TNF-α−) and # indicates *p* < 0.05 versus TNF-α alone (PA 0 µM, TNF-α+) (Šídák-adjusted). Only significant comparisons are annotated.

As we observed that PA and PA + TNF-α induced apoptotic cell death, we next evaluated their effects on cell-cycle distribution using propidium iodide (PI) staining and flow cytometry. NSCLC cells were treated with PA alone or in combination with TNF-α for 24 h. In H1299 cells, PA (5, 7.5, and 10 µM) significantly increased the G1-phase population (Figure 6A,B), with a concomitant reduction in the S- and G2/M-phase fractions (Figure 6A,B). TNF-α monotherapy similarly increased the G1-phase fraction (Figure 6A,B). Co-treatment with PA + TNF-α produced a G1-arrest profile broadly comparable to TNF-α alone; however, based on the quantified sub-G1 percentages from three independent experiments (*n* = 3), the combination of 10 µM PA with TNF-α significantly increased the sub-G1 (apoptotic) population compared with untreated cells (Figure 6A,B; * *p* < 0.05). In A549 cells, PA (5, 7.5, and 10 µM) or TNF-α alone increased the G1-phase fraction (Figure 6C,D). Notably, PA + TNF-α significantly elevated the sub-G1 fraction versus untreated control at 7.5 and 10 µM PA (Figure 6C,D; * *p* < 0.05), indicating that the combination increases apoptotic (sub-G1) accumulation while maintaining an overall G1-enriched distribution.

**Figure 7:**
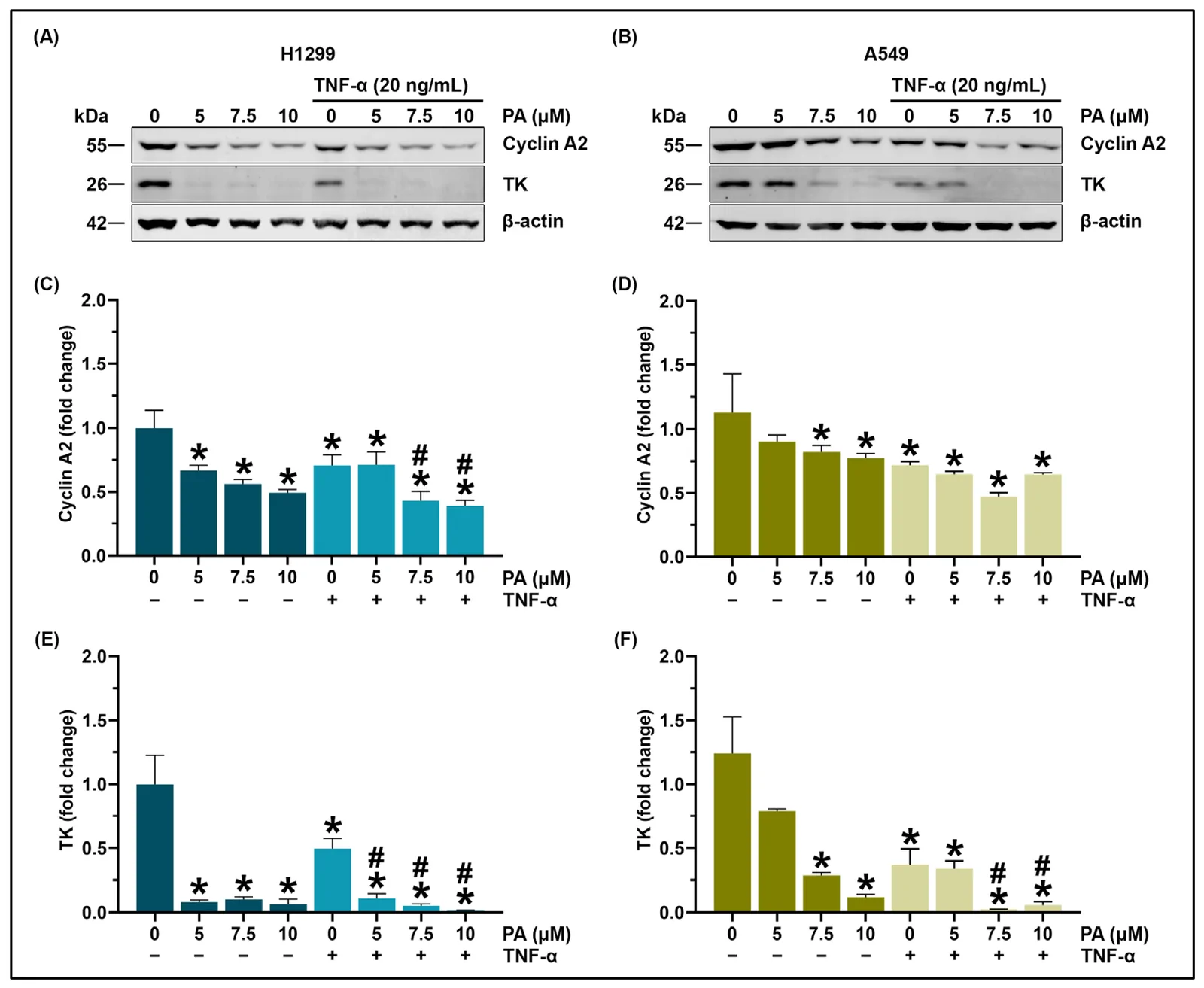
Effects of PA and PA + TNF-α on cell-cycle regulatory proteins in H1299 and A549 cells. Representative Western blot images showing cyclin A2 and thymidine kinase (TK) expression in H1299 (**A**) and A549 (**B**) cells following the indicated treatments. β-actin served as the loading control and was used for normalization. Densitometric quantification is shown for cyclin A2 in H1299 (**C**) and A549 (**D**), and TK in H1299 (**E**) and A549 (**F**). Data are presented as mean ± SEM from *n* = 3 independent biological replicates per condition. Statistical analyses were performed separately for each cell line using ordinary two-way ANOVA (α = 0.05) with factors PA concentration (0, 5, 7.5, and 10 µM) and TNF-α treatment (±TNF-α), followed by Šídák’s multiple-comparisons test to compare (i) each treatment versus the untreated control and (ii) PA + TNF-α versus TNF-α alone at matched PA concentrations. In the graphs, * indicates *p* < 0.05 versus the untreated control and # indicates *p* < 0.05 versus TNF-α alone (multiplicity-adjusted); unmarked comparisons are not significant (ns). The original Western blot images can be found in the Supplementary Materials.

To substantiate the PI-based cell-cycle findings with molecular evidence, we evaluated cyclin A2 and thymidine kinase (TK), two well-established markers of G1/S transition and S-phase progression. Cyclin A2 is induced at the G1/S boundary and during S phase to support DNA replication, whereas TK is an S-phase-associated enzyme linked to nucleotide metabolism and is widely used as a surrogate indicator of DNA synthesis activity. In H1299 cells, PA (5–10 µM) reduced cyclin A2 expression compared with the untreated control (Figure 7A,C; * *p* < 0.05). TNF-α alone also decreased cyclin A2 levels. Co-treatment with TNF-α and PA further decreased cyclin A2 relative to TNF-α alone at the PA concentrations showing a significant # comparison (Figure 7A,C; # *p* < 0.05), consistent with enhanced suppression of S-phase-associated cyclin A2 under combination treatment. In A549 cells, PA (7.5–10 µM) and TNF-α similarly decreased cyclin A2 compared with the control (Figure 7B,D; * *p* < 0.05), and the PA + TNF-α combination produced an additional reduction versus TNF-α alone where significant (Figure 7B,D; # *p* < 0.05). Across the tested PA concentration range, cyclin A2 levels decreased progressively with increasing PA dose, supporting a dose-gradient suppression of an S-phase-associated cyclin.

We next assessed TK protein expression. In H1299 cells, PA (5, 7.5, and 10 µM) and TNF-α alone decreased TK compared with the untreated control (Figure 7A,E; * *p* < 0.05). Combination treatment reduced TK further versus TNF-α alone at the PA concentrations with significant # comparisons (Figure 7A,E; # *p* < 0.05). Similarly, in A549 cells, PA and TNF-α decreased TK expression relative to the control (Figure 7B,F; *p < 0.05), and co-treatment with PA + TNF-α further reduced TK compared with TNF-α alone at the doses showing significance (Figure 7B,F; # *p* < 0.05). Notably, TK expression also showed a dose-gradient reduction across 5–10 µM PA, consistent with concentration-dependent suppression of an S-phase entry/replication marker. Overall, these data indicate that PA, particularly in combination with TNF-α, downregulates S-phase-associated proteins, supporting the cell-cycle redistribution observed by flow cytometry.

**Figure 8:**
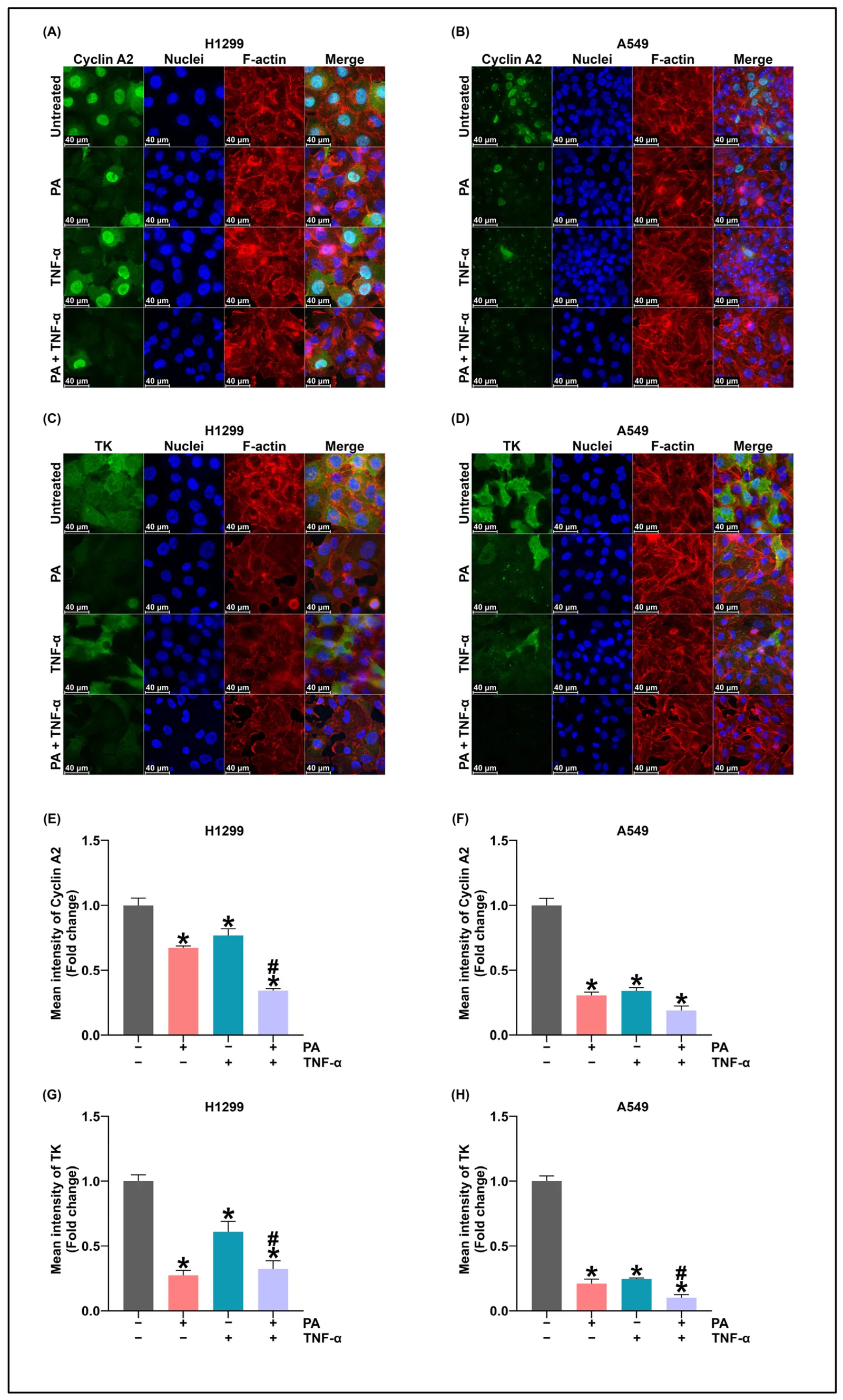
Immunofluorescence analysis of cyclin A2 and thymidine kinase (TK) following PA and TNF-α treatment in H1299 and A549 cells. Representative immunofluorescence images of cyclin A2 in H1299 (**A**) and A549 (**B**) cells, and TK in H1299 (**C**) and A549 (**D**) cells after 24 h of treatment with PA (10 µM) and/or TNF-α (20 ng/mL). Quantification of cyclin A2 background-corrected mean fluorescence intensity per cell is shown for H1299 (**E**) and A549 (**F**), and quantification of TK background-corrected mean fluorescence intensity per cell is shown for H1299 (**G**) and A549 (**H**). Cyclin A2 and TK are shown in green; nuclei were counterstained with DAPI (blue), and F-actin was labeled with phalloidin (red). Images were acquired using a 100× oil-immersion objective under identical imaging settings for all treatment groups within each experiment. Brightness and contrast adjustments, where applied for figure preparation, were performed uniformly across treatment groups within the same staining experiment. For each biological replicate, at least 50 cells in total, sampled across five randomly selected fields, were quantified and averaged to generate a single value per condition. Fluorescence-intensity values were normalized to the untreated control (UT = 1.0). Data are presented as mean ± SEM from *n* = 3 independent biological replicates per condition. Statistical analyses were performed using one-way ANOVA (α = 0.05) followed by Tukey’s multiple-comparisons test. * *p* < 0.05 versus the untreated control and # indicates *p* < 0.05 versus TNF-α alone; unmarked comparisons were not significant.

To visualize the expression and intracellular localization of these two markers, we performed an immunofluorescence analysis of cyclin A2 and thymidine kinase (TK) following 24 h of treatment with TNF-α (20 ng/mL), PA (10 µM), or their combination (Figure 8). Fluorescence signals were quantified as background-corrected mean fluorescence intensity on a per-cell basis. In H1299 cells, TNF-α, PA, and PA + TNF-α each significantly reduced cyclin A2 and TK fluorescence intensity compared with the untreated controls (Figure 8A,C,E,G; * *p* < 0.05). Consistent with the results from protein analysis (Figure 7), the combination treatment significantly reduced cyclin A2 and TK fluorescence intensity (Figure 8E,G). Although residual TK staining remained visually detectable in some individual H1299 cells, quantitative analysis demonstrated a significant reduction in background-corrected mean TK fluorescence intensity, consistent with the reduction in population-average TK protein abundance observed by immunoblotting. Similarly, in A549 cells, TNF-α, PA, and PA + TNF-α each attenuated cyclin A2 and TK fluorescence intensity (Figure 8B,D,F,H; * *p* < 0.05). Combination treatment significantly decreased TK fluorescence intensity in A549 cells. Overall, the immunofluorescence findings are consistent with the immunoblot data in showing that PA and/or TNF-α reduce cyclin A2 and TK expression.

### 3.5. In Silico ADME and Drug-Likeness Analysis of PA

To evaluate the translational potential and drug-likeness of panduratin A (PA), an in silico ADME analysis was performed using the SwissADME platform (Table 2). The predicted physicochemical profile indicated that PA has a molecular weight of 406.51 g/mol, a topological polar surface area (TPSA) of 66.76 Å^2^, and two hydrogen bond donors alongside four hydrogen bond acceptors. PA demonstrated high gastrointestinal (GI) absorption without blood–brain barrier (BBB) permeation, suggesting a lower likelihood of central nervous system exposure. Furthermore, PA showed an overall favorable drug-likeness profile, while it met Lipinski criteria (zero violations) and Veber rules (Table 2) and yielded a bioavailability score of 0.55. However, PA was predicted to be poorly soluble across multiple models (ESOL, Ali, and SILICOS-IT) and showed high lipophilicity (consensus Log P*_o/w_* = 5.19), which may limit developability and necessitate formulation strategies. In addition, PA did not satisfy Ghose, Egan, and Muegge filters due to lipophilicity-related thresholds and was not lead-like (two violations: MW > 350 and XLOGP3 > 3.5). These findings suggest favorable oral absorption potential of PA, while highlighting solubility and lipophilicity as key liabilities to address in future optimization and formulation studies.

## 4. Discussion

Discovery and development of improved anticancer approaches remain important to address acquired resistance and the limitations of conventional treatments. In this study, we used an in vitro NSCLC cell model to investigate the anticancer effects of panduratin A (PA) and to examine whether PA can sensitize non-small-cell lung-cancer (NSCLC) cells to TNF-α-associated apoptotic cell death. We found that PA reduced cell number (cells/field), consistent with impaired cellular fitness, and induced cell death; moreover, co-treatment with TNF-α further enhanced apoptosis in NSCLC cells under the experimental conditions tested. The pro-apoptotic activity of PA was supported by increased activation (cleavage) of caspase-3 and processing of PARP-1, consistent with engagement of a caspase-dependent apoptotic program. In parallel, PA increased the G1-phase fraction, indicating a G1-associated cell-cycle arrest phenotype, and PA + TNF-α increased the sub-G1 population, consistent with enhanced apoptotic accumulation under the same treatment conditions while retaining an overall G1-associated distribution. In addition, TNF-α alone significantly reduced the cell number of both H1299 and A549 cells, confirming measurable TNF-α responsiveness in our in vitro system.

Typically, TNF-α is a pleiotropic cytokine that can simultaneously activate both pro-survival and pro-apoptotic signaling pathways [30]. A major pro-survival branch is mediated, in part, by activation of NF-κB following binding of TNF-α to TNFR [34,38,42]. Numerous studies have shown that inhibition of NF-κB signaling can sensitize cancer cells to cytotoxic stimuli, including chemotherapy [38,43,44]. Prior studies have demonstrated that PA suppresses NF-κB activity in multiple cell types, including those of lung cancer origin [11,14,17]. Therefore, the combination of PA and TNF-α could enhance cell death compared to treatment with either agent alone. Accordingly, one plausible explanation for the enhanced cell death observed with PA + TNF-α is that PA may attenuate TNF-α-induced pro-survival signaling (e.g., NF-κB), thereby shifting the overall TNF-α response toward apoptosis; this proposed mechanism requires direct validation in our experimental system. Importantly, to move beyond groupwise statistical comparisons, we applied a quantitative drug-interaction framework (Bliss independence model; Table 1). This analysis supports that the PA + TNF-α combination can produce effects greater than expected under additivity, consistent with a synergistic interaction under our experimental conditions. Together, these findings strengthen the conclusion that PA not only exhibits anticancer activity as a single agent but can also function as a sensitizing partner that enhances TNF-α-associated cytotoxicity in NSCLC cells.

Specifically, since apoptotic cell death was associated with caspase and PARP-1 activation [45,46,47,48], our results demonstrated that the combination of PA and TNF-α significantly enhanced the cleavage of executioner caspase-3 as well as its major target—PARP-1, leading to increased apoptotic cell death. Consistent with an apoptotic mechanism, the observed increases in cleaved caspase-3 and cleaved PARP-1 with combination treatment provide biochemical support for a caspase-dependent basis of the enhanced apoptotic phenotype. In A549 cells, the greater PARP-1 cleavage observed with PA + TNF-α than with TNF-α alone supports a stronger biochemical apoptotic response to the combination treatment. However, because the PARP-1-cleavage signal with TNF-α alone was comparatively limited in the representative immunoblot, the effect of TNF-α alone on PARP-1 cleavage should be interpreted cautiously. Because our study used two NSCLC models with distinct genetic backgrounds, including H1299 (p53-null) and A549 (p53 wild-type) [49,50], differences in checkpoint and stress-response signaling may contribute to the cell-line-dependent magnitude of the response observed in Figure 3 [51,52]. It should be noted that Figure 3 quantifies the apoptotic fraction by flow cytometry at a defined time point, whereas the Hoechst-based cell-count assay reports endpoint cell number/field and therefore integrates cumulative effects of both proliferation and cell loss over the treatment period. Accordingly, similar endpoint cell numbers can still be observed even if the apoptotic fraction differs, particularly if baseline proliferation rates and/or treatment-induced growth arrest differ between cell lines. Notably, even when the combination treatment produces a comparable reduction in overall cell number at the endpoint, upstream apoptotic phenotypes can differ in magnitude between cell lines due to differences in apoptotic priming and pathway balance. While p53 status can modulate transcriptional programs linked to cell-cycle arrest and apoptosis, additional targeted experiments would be required to establish a direct causal role of p53 in the PA/TNF-α responses observed here. Notably, we also observed that TNF-α alone induced a higher apoptotic fraction in A549 cells than in H1299 cells under identical exposure conditions, suggesting baseline cell-line-dependent differences in TNF-α sensitivity that may reflect differences in apoptotic priming and/or TNF-α-activated pro-survival signaling.

Additionally, we observed that PA induced cell-cycle arrest. Cell-cycle arrest is a critical regulatory mechanism that occurs in response to cellular stress or DNA damage, preventing the propagation of compromised cells by suspending the division process [53,54,55]. Apoptosis and cell-cycle arrest are intrinsically linked, as prolonged cell-cycle arrest often transitions into apoptosis if the underlying damage is irreparable [56,57,58]. PA exhibits cell-specific modulation of the cell-cycle, triggering G0/G1-phase arrest in breast cancer (MCF-7) while promoting G2/M-phase arrest in prostate (PC3) and lung (A549) carcinoma models [11,16,18]. However, in this study, we found that PA induced a G1-associated shift in cell-cycle distribution that was accompanied by downregulating the expression of thymidine kinase and cyclin A2. Given that thymidine kinase is critical for DNA synthesis during the G1 to S-phase progression [59,60], its suppression by PA provides a mechanistic basis for the observed G1-phase arrest. In line with these results, PA treatment downregulated cyclin A2. G1-phase arrest is often accompanied by suppression of the S-phase initiation program, including reduced expression of E2F-regulated targets such as cyclin A2, which is essential for S-phase progression [61,62,63]. Consequently, the observed reduction in cyclin A2 levels further substantiates the induction of G1-phase arrest in NSCLC cells. The divergent results regarding the cell-cycle phase between our findings and those of the previous study [11] may stem from differences in the PA dosages employed, cyclin regulation, genetic background, and analytical methodologies. In our experimental design, we evaluated the concentrations of PA (5, 7.5, and 10 µM). Across this range, PA induced a dose-dependent G1-phase arrest in both A549 (p53 wild-type) and H1299 (p53-null) NSCLC cells. Consistent with this phenotype, western blot analysis demonstrated a clear concentration-dependent (stepwise) reduction in cyclin A2 and thymidine kinase (TK) with increasing PA dose, supporting suppression of the G1/S transition and S-phase entry. This G1 blockade is further supported by our immunofluorescence data, which revealed reduced cyclin A2 and thymidine kinase (TK), which are key drivers required for DNA replication and S-phase progression. The consistent G1-arrest observed in both p53 wild-type and p53-null cells suggests that PA can promote G1 accumulation in both p53 wild-type and p53-null contexts under these conditions, although the upstream regulatory mechanisms remain to be defined, at subcytotoxic to moderate pharmacological doses (5–10 µM). Conversely, the previously reported G2/M arrest utilized a higher concentration (5 µg/mL or 12.3 µM). Furthermore, methodological distinctions such as the previous study’s use of an in-house plant fraction (>98% purity) coupled with mitosis-specific Phospho-Histone H3 staining versus our commercial standard (Biosynth) and PI DNA flow cytometry likely amplified these phenotypic differences, and support the possibility that PA-induced cell-cycle effects may be dose- and context-dependent.

Based on the in silico ADME predictions, PA is predicted to have high gastrointestinal absorption and no blood–brain barrier permeation. However, the predicted high lipophilicity and poor solubility indicate potential developability liabilities that may require formulation strategies and/or optimization in future studies.

Importantly, the present work was intentionally designed as an in vitro model to establish proof-of-concept evidence that PA can modulate TNF-α-associated cytotoxic responses and to identify measurable molecular correlates (caspase-3/PARP-1 processing, cell-cycle redistribution, and downregulation of cyclin A2 and TK) that can be carried forward into more disease-relevant systems. Accordingly, several limitations should be considered. First, our findings are based on two NSCLC cell lines representing adenocarcinoma (A549) and large-cell carcinoma (H1299); therefore, generalizability to other NSCLC contexts—including squamous cell carcinoma and genotype-defined subtypes such as EGFR-mutant or ALK-rearranged NSCLC—remains to be established and may limit the universality of the conclusions. Second, we did not evaluate toxicity in non-malignant lung epithelial cells (e.g., BEAS-2B); therefore, tumor selectivity and potential cytotoxicity to normal cells could not be assessed, and future safety studies are needed to determine the therapeutic window and clinical translational potential. Third, the proposed involvement of TNF-α-associated survival signaling (such as NF-κB) was inferred from prior literature and not directly evaluated in our system; future studies should quantify pathway activity and test causality using pharmacologic or genetic approaches. Moreover, NF-κB pathway involvement was not directly verified in this study (e.g., by assessing p65 phosphorylation/nuclear translocation or NF-κB reporter activity), and therefore this mechanistic link should be considered hypothesis-generating until confirmed. In addition, cell-death readouts can be influenced by changes in cell number/proliferation; therefore, complementary cell-counting measurements provide an orthogonal measure of reduced cellular fitness. Finally, these findings require validation in more complex, disease-relevant models (e.g., 3D spheroids, co-culture/immune-competent systems, patient-derived models, and in vivo studies) to determine whether the observed molecular responses translate to antitumor activity, exposure feasibility, and acceptable safety.

**Figure 9:**
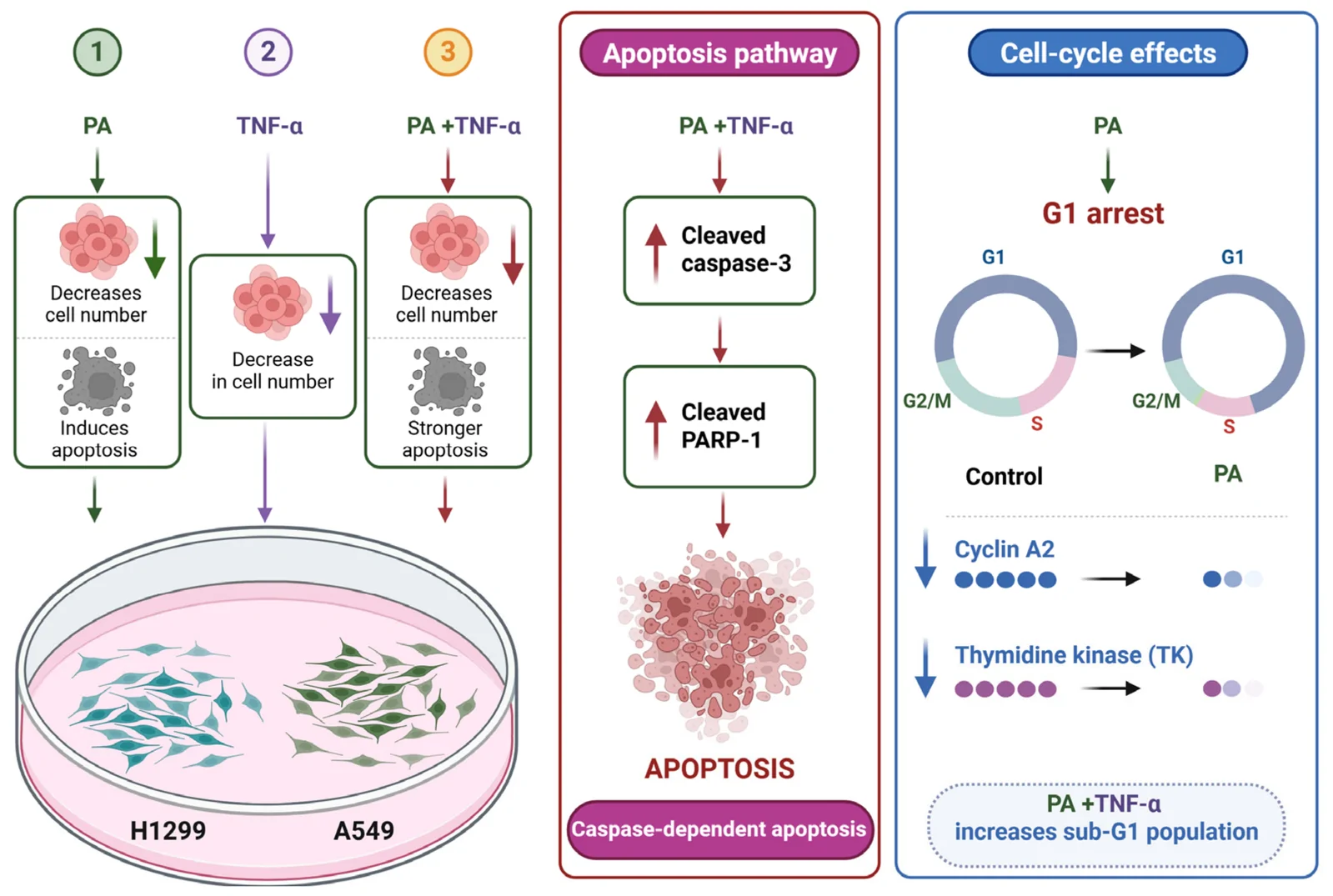
Proposed working model summarizing the key effects of panduratin A (PA) alone and in combination with TNF-α in NSCLC cells. In H1299 and A549 cells, PA reduced cellular fitness as reflected by decreased cell number and promoted apoptotic cell death. PA-induced apoptosis was associated with increased executioner caspase-3 activation (cleavage) and PARP-1 processing, consistent with a caspase-dependent apoptotic program. PA also induced a G1-associated cell-cycle arrest phenotype, accompanied by downregulation of G1/S-associated markers cyclin A2 and thymidine kinase (TK). TNF-α alone produced a measurable reduction in cell number under the conditions tested, and co-treatment with PA further enhanced TNF-α-associated cytotoxic readouts, including increased apoptotic accumulation (sub-G1). Created in BioRender. Nimlamool, W. (2026) https://BioRender.com/cp9yisu, accessed on 27 August 2026.

Collectively, our in vitro data provide a mechanistic framework supporting PA as a candidate modulator of TNF-α-associated apoptosis and cell-cycle control in NSCLC cells, as summarized schematically in Figure 9. By establishing these phenotypic and molecular readouts in a controlled cell-based system, our findings provide a rationale for future studies to (i) validate pathway-level mechanisms, (ii) formalize drug-interaction relationships, and (iii) extend evaluation into disease-relevant and in vivo models before any clinical implications can be drawn.

## 5. Conclusions

This study establishes an in vitro framework showing that panduratin A (PA) suppresses NSCLC cell fitness by reducing cell number and promoting apoptotic cell death in both H1299 and A549 cells. PA-induced apoptosis was supported by increased executioner caspase-3 activation and PARP-1 processing, indicating engagement of a caspase-dependent apoptotic program. In parallel, PA perturbed cell-cycle progression by increasing the G1-phase fraction and downregulating G1/S-associated markers, including thymidine kinase (TK) and cyclin A2, in a concentration-dependent manner, consistent with a G1-associated arrest phenotype. Under the same experimental conditions, co-treatment with TNF-α increased apoptotic accumulation and further intensified cell-death-associated readouts compared with single-agent exposure, supporting the concept that PA can enhance TNF-α-associated cytotoxic readouts under these conditions. Collectively, our results provide proof-of-concept evidence that PA induces apoptosis and a G1-associated cell-cycle phenotype while enhancing TNF-α-associated cytotoxicity in NSCLC cells. These data support the use of PA-based combinations as a testable strategy for further mechanistic investigation and preclinical model extension, providing a foundation for future in vivo and disease-relevant studies.

## Figures and Tables

**Table 1:** Bliss independent model.

H1299								
PA (µM)	Cell Count (Cells/Field)	Fractional Inhibition	Treatment		Cell Count (Cells/Field)	Fractional Inhibition	Bliss Expected	Bliss Score
			TNF-α (ng/mL)	PA (µM)				
0.00	11,689.5	0.000	20	0.00	7579.5	0.352	0.352	0.000
0.16	12,057.0	−0.031	20	0.16	7914.0	0.323	0.331	−0.008
0.31	12,523.5	−0.071	20	0.31	7956.0	0.319	0.305	0.014
0.63	12,966.5	−0.109	20	0.63	7584.5	0.351	0.281	0.070
1.25	13,708.0	−0.173	20	1.25	7523.0	0.356	0.240	0.117
2.50	12,552.5	−0.074	20	2.50	7210.5	0.383	0.304	0.079
5.00	7002.0	0.401	20	5.00	2523.0	0.784	0.612	0.173
10.00	3812.0	0.674	20	10.00	1429.5	0.878	0.789	0.089
20.00	1602.0	0.863	20	20.00	823.0	0.930	0.911	0.018
40.00	23.50	0.998	20	40.00	20.0	0.998	0.999	0.000
**A549**								
**PA (µM)**	**Cell Count (Cells/Field)**	**Fractional Inhibition**	**Treatment**		**Cell Count (Cells/Field)**	**Fractional Inhibition**	**Bliss Expected**	**Bliss Score**
			**TNF-α (ng/mL)**	**PA (µM)**				
0.00	11,402.5	0.000	20	0.00	7441.5	0.347	0.347	0.000
0.16	11,766.0	−0.032	20	0.16	7788.0	0.317	0.327	−0.010
0.31	12,270.0	−0.076	20	0.31	7824.5	0.314	0.298	0.016
0.63	12,572.0	−0.103	20	0.63	7492.5	0.343	0.280	0.062
1.25	13,288.5	−0.165	20	1.25	7447.0	0.347	0.239	0.107
2.50	12,241.5	−0.074	20	2.50	7117.0	0.376	0.299	0.076
5.00	6951.5	0.390	20	5.00	2519.0	0.779	0.602	0.177
10.00	3759.5	0.670	20	10.00	1419.0	0.876	0.785	0.091
20.00	1563.0	0.863	20	20.00	797.0	0.930	0.911	0.020
40.00	15.0	0.999	20	40.00	15.0	0.999	0.999	0.000

**Table 2:** Predicted in silico ADME, pharmacokinetic, and drug-likeness properties of panduratin A using the SwissADME platform.

Category	Property/Parameter	Predicted Value
Physicochemical Properties	Molecular Weight (g/mol)	406.51
	Heavy Atoms	30
	Aromatic Heavy Atoms	12
	Fraction Csp3	0.35
	Rotatable Bonds	6
	H-bond Acceptors	4
	H-bond Donors	2
	Molar Refractivity	121.48
	TPSA (A°2)	66.76
Lipophilicity	Consensus Log *P_o/w_*	5.19
Water Solubility	ESOL Class	Poorly soluble
Pharmacokinetics	GI Absorption	High
	BBB Permeation	No
	P-gp Substrate	No
	Log *K_p_* (Skin Permeation, cm/s)	−4.55
Drug-Likeness	Lipinski Rule	Yes; zero violations
	Ghose Rule	No; one violation: WLOGP > 5.6
	Veber Rule	Yes
	Egan Rule	No; one violation: WLOGP > 5.88
	Muegge Rule	No; one violation: XLOGP3 > 5
	Bioavailability Score	0.55
Medicinal Chemistry	PAINS Alerts	zero alerts
	Brenk Alerts	one alert (alkene)
	Leadlikeness Violation	No; (two violations: MW > 350, XLOGP3 > 3.5)
	Synthetic Accessibility Score	4.41

Abbreviations: TPSA, Topological Polar Surface Area; $\mathrm{Log}\;P_{o/w}$, octanol-water partition coefficient; GI, Gastrointestinal; BBB, Blood–Brain Barrier; P-gp, P-glycoprotein; CYP, Cytochrome P450; $\mathrm{Log}\;K_{p}$, skin permeation coefficient; PAINS, Pan-Assay Interference Compounds. (Access date: 13 August 2026).

## Data Availability

The original contributions presented in this study are included in the article. Further inquiries can be directed to the corresponding author.

## Supplementary Materials

The following supporting information can be downloaded at: https://www.mdpi.com/article/10.3390/biom16091312/s1, Method S1: Bliss-independent model calculation; Method S2: In silico ADME and Druglikeness Analysis; Table S1: Bliss Independent Model; Table S2: Predicted in silico ADME, pharmacokinetic, and drug-likeness properties of panduratin A using the SwissADME platform. The original Western blot images can be found in this section.
