# Supplementary material for: Panduratin A Induces Caspase-Dependent Apoptosis and G1-Associated Cell-Cycle Arrest and Enhances TNF-α-Associated Cytotoxicity in NSCLC Cells
Source: Biomolecules. 2026 Sep 10;16(9):1312. doi: 10.3390/biom16091312 (PMC13604354; doi:10.3390/biom16091312)
Supplement: Supplementary file 1 [file biomolecules-16-01312-s001.zip › biomolecules-4484757-Original Western blot/Figure S2A.pdf]

Figure 7A Effect of PA and PA+TNF-α on protein expression of cell-cycle markers in H1299 cells

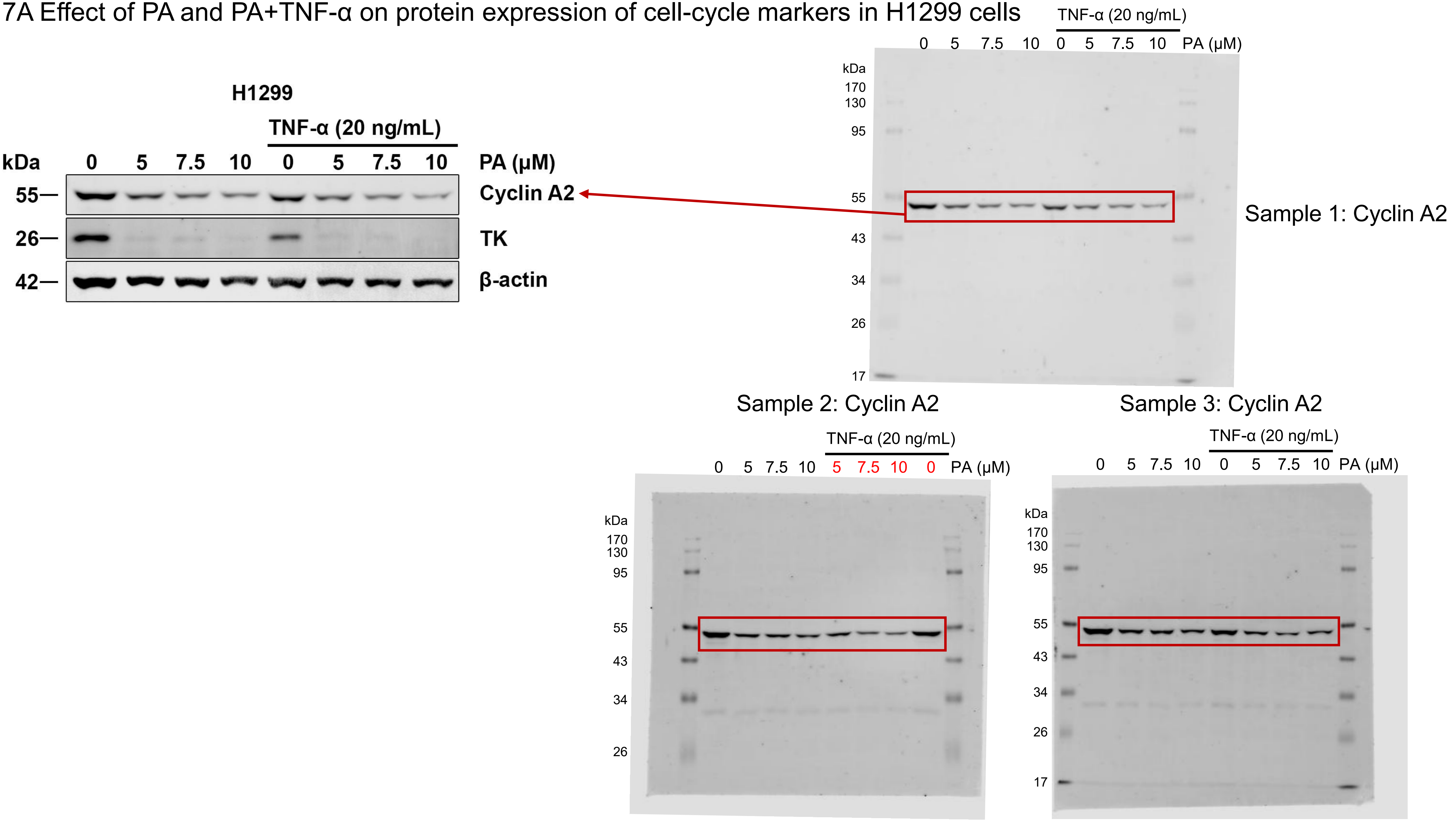

Thermo Scientific PageRuler Prestained Protein Ladder Pub. No. MAN0011772 Assembling Lot 00702236 (Cat. No. 26616)

Figure 7A Effect of PA and PA+TNF-α on protein expression of cell-cycle markers in H1299 cells

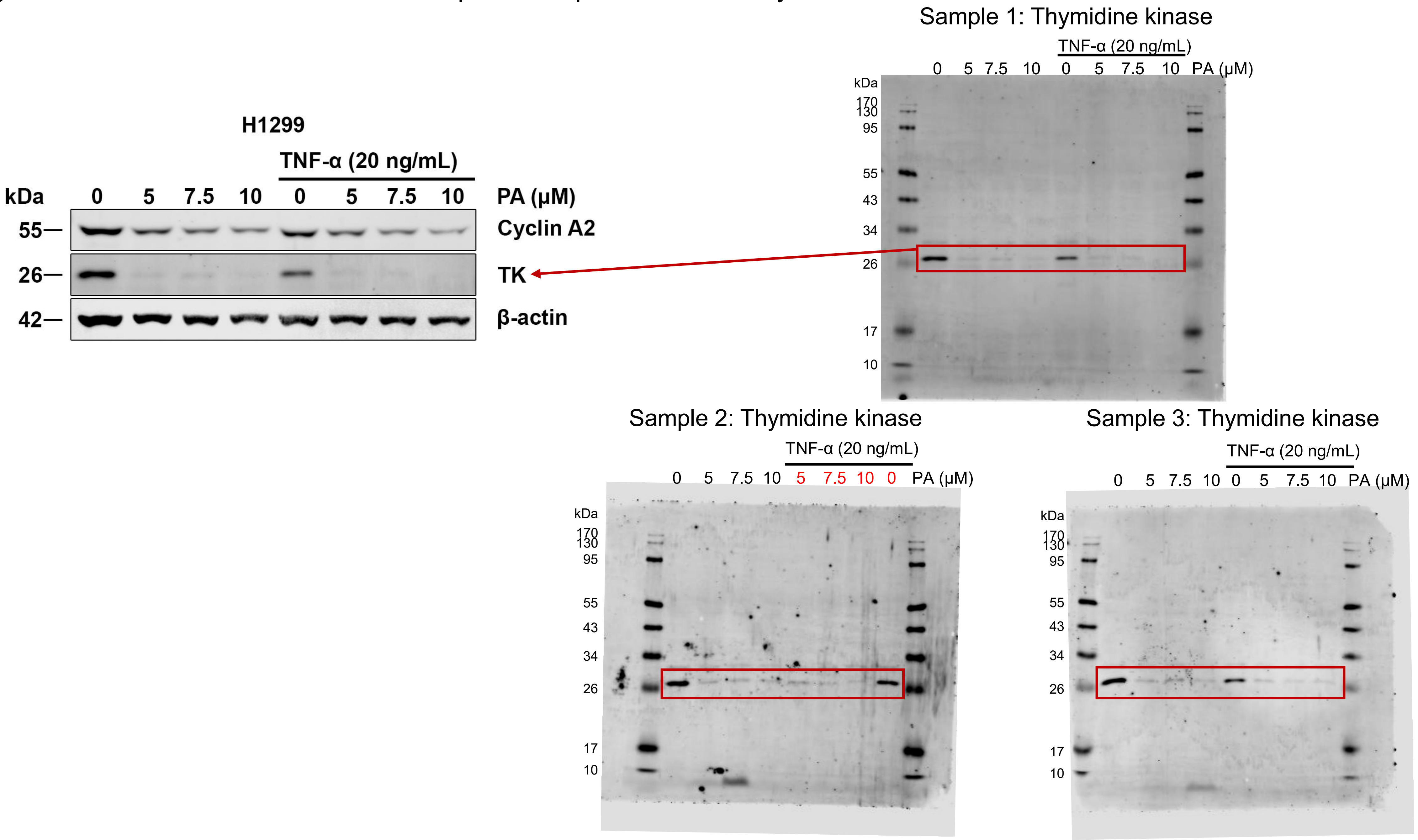

Thermo Scientific PageRuler Prestained Protein Ladder Pub. No. MAN0011772 Assembling Lot 00702236 (Cat. No. 26616)

Figure 7A Effect of PA and PA+TNF-α on protein expression of cell-cycle markers in H1299 cells

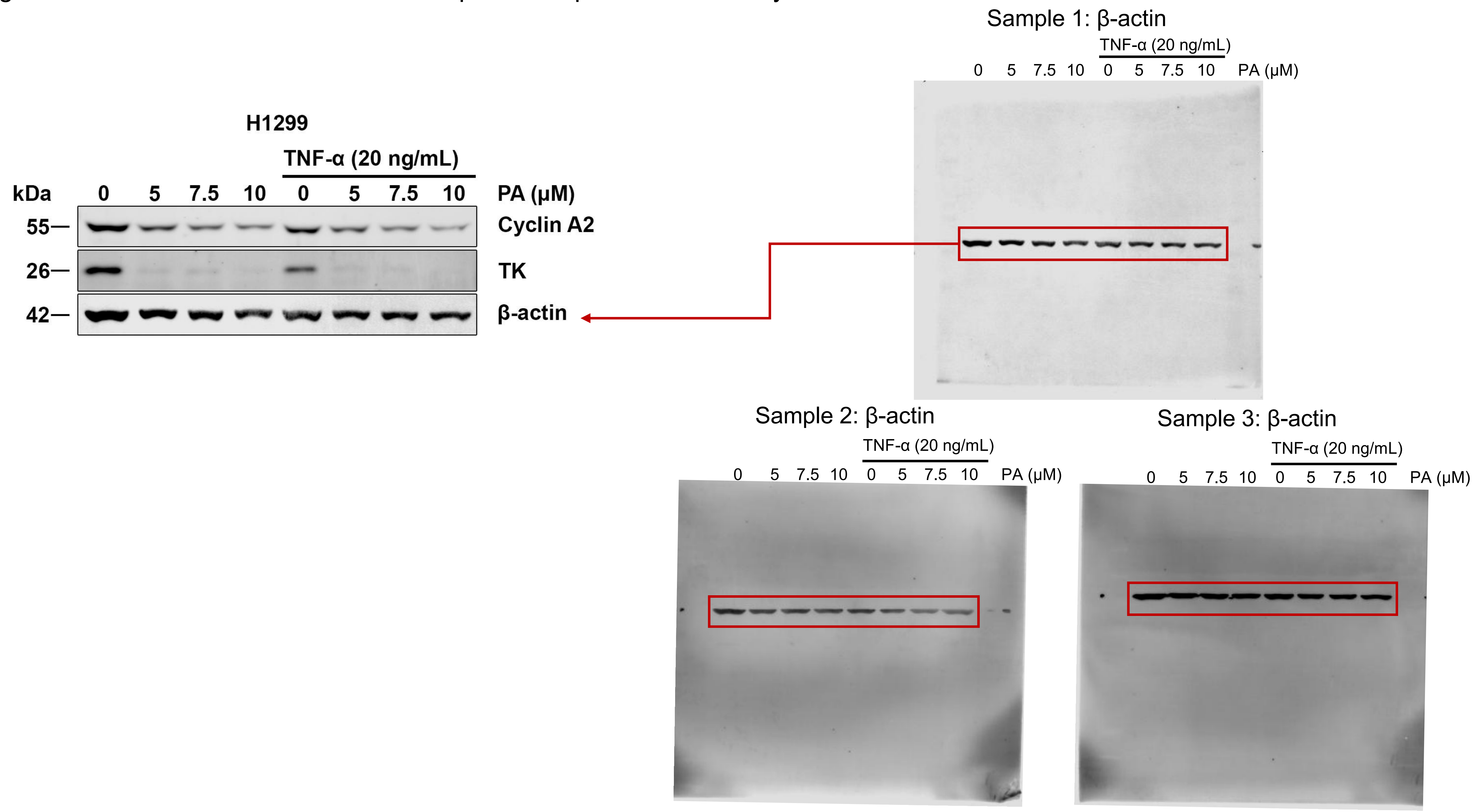

Thermo Scientific PageRuler Prestained Protein Ladder Pub. No. MAN0011772 Assembling Lot 00702236 (Cat. No. 26616)
