# Supplementary material for: Panduratin A Induces Caspase-Dependent Apoptosis and G1-Associated Cell-Cycle Arrest and Enhances TNF-α-Associated Cytotoxicity in NSCLC Cells
Source: Biomolecules. 2026 Sep 10;16(9):1312. doi: 10.3390/biom16091312 (PMC13604354; doi:10.3390/biom16091312)
Supplement: Supplementary file 1 [file biomolecules-16-01312-s001.zip › biomolecules-4484757-supplementary.pdf]

Supplementary Materials

Method S1 Bliss-independent model calculation

The interaction between PA and TNF-α was evaluated using the Bliss independence model. After obtaining cell numbers following treatment with PA, TNF-α, and PA + TNF-α, fractional inhibition values were calculated for PA (E_P), TNF-α (E_T), and the combination (E_{PT}) relative to the untreated control. Negative fractional inhibition values indicate cell numbers slightly above control and were retained as observed values in the Bliss calculation. The Bliss-expected combined inhibition (E_{exp}) was calculated as:

$$E_{\text{exp}} = E_P + E_T - (E_P \times E_T).$$

The Bliss score was then calculated as:

$$\text{Bliss score} = E_{PT} - E_{\text{exp}}.$$

A positive Bliss score indicates synergy, a value near 0 indicates additivity, and a negative value indicates antagonism.

Results

Table S1 Bliss Independent Model

H1299								
PA (μM)	cell count (cells/field)	Fractional inhibition	Treatment		cell count (cells/field)	Fractional inhibition	Bliss expected	Bliss score
			TNF-α (ng/mL)	PA (μM)				
0.00	11,689.5	0.000	20	0.00	7,579.5	0.352	0.352	0.000
0.16	12,057.0	-0.031	20	0.16	7,914.0	0.323	0.331	-0.008
0.31	12,523.5	-0.071	20	0.31	7,956.0	0.319	0.305	0.014
0.63	12,966.5	-0.109	20	0.63	7,584.5	0.351	0.281	0.070
1.25	13,708.0	-0.173	20	1.25	7,523.0	0.356	0.240	0.117
2.50	12,552.5	-0.074	20	2.50	7,210.5	0.383	0.304	0.079
5.00	7,002.0	0.401	20	5.00	2,523.0	0.784	0.612	0.173
10.00	3,812.0	0.674	20	10.00	1,429.5	0.878	0.789	0.089
20.00	1,602.0	0.863	20	20.00	823.0	0.930	0.911	0.018
40.00	23.50	0.998	20	40.00	20.0	0.998	0.999	0.000
A549								
PA (μM)	cell count (cells/field)	Fractional inhibition	Treatment		cell count (cells/field)	Fractional inhibition	Bliss expected	Bliss score
			TNF-α (ng/mL)	PA (μM)				
0.00	11,402.5	0.000	20	0.00	7,441.5	0.347	0.347	0.000
0.16	11,766.0	-0.032	20	0.16	7,788.0	0.317	0.327	-0.010
0.31	12,270.0	-0.076	20	0.31	7,824.5	0.314	0.298	0.016
0.63	12,572.0	-0.103	20	0.63	7,492.5	0.343	0.280	0.062
1.25	13,288.5	-0.165	20	1.25	7,447.0	0.347	0.239	0.107
2.50	12,241.5	-0.074	20	2.50	7,117.0	0.376	0.299	0.076
5.00	6,951.5	0.390	20	5.00	2,519.0	0.779	0.602	0.177
10.00	3,759.5	0.670	20	10.00	1,419.0	0.876	0.785	0.091
20.00	1,563.0	0.863	20	20.00	797.0	0.930	0.911	0.020
40.00	15.0	0.999	20	40.00	15.0	0.999	0.999	0.000

Method S2 In silico ADME and Druglikeness Analysis

To predict the pharmacokinetic profile and drug-likeness of panduratin A (PA), in silico ADME (absorption, distribution, metabolism, and excretion) profiling was performed using the SwissADME web server (https://www.swissadme.ch/; accessed 13 August 2026). The canonical SMILES of PA (PubChem CID: 6483648) was retrieved from the PubChem database and submitted to SwissADME. Key physicochemical descriptors were calculated, including molecular weight (MW), topological polar surface area (TPSA), number of rotatable bonds, hydrogen-bond donors and acceptors, and lipophilicity (consensus Log $P_{o/w}$). Predicted pharmacokinetic parameters included gastrointestinal (GI) absorption, blood–brain barrier (BBB) permeation, P-glycoprotein (P-gp) substrate status, and skin permeation (log *K*p). Drug-likeness and medicinal chemistry properties were assessed using the Lipinski, Ghose, Veber, Egan, and Muegge filters, along with the bioavailability score, PAINS alerts, Brenk structural alerts, lead-likeness evaluation, and synthetic accessibility.

Results

Table S2. Predicted in silico ADME, pharmacokinetic, and drug-likeness properties of panduratin A using the SwissADME platform.

(Access date: 13 August 2026)

SMILES: COc1cc(O)c(c(c1)O)C(=O)[C@@H]1[C@H](CC=C(C)C)C(=CC[C@H]1c1ccccc1)C

Category	Property / Parameter	Predicted Value
Physicochemical Properties	Formula	C26H30O4
	Molecular Weight	406.51 g/mol
	Num. heavy atoms	30
	Num. arom. heavy atoms	12
	Fraction Csp3	0.35
	Num. rotatable bonds	6
	Num. H-bond acceptors	4
	Num. H-bond donors	2
	Molar Refractivity	121.48
	TPSA	66.76 Å²
Lipophilicity	Log $P_{o/w}$ (XLOGP3)	5.96
	Log $P_{o/w}$ (WLOGP)	6.01
	Log $P_{o/w}$ (MLOGP)	3.59
	Log $P_{o/w}$ (SILICOS-IT)	5.20
	Consensus Log $P_{o/w}$	5.19
Water Solubility	Log S (ESOL)	-6.02

Category	Property / Parameter	Predicted Value
	Solubility	3.93e-04 mg/mL ; 9.66e-07 mol/L
	Class	Poorly soluble
	Log S (Ali)	-7.14
	Solubility	2.96e-05 mg/mL ; 7.28e-08 mol/L
	Class	Poorly soluble
	Log S (SILICOS-IT)	-6.14
	Solubility	2.94e-04 mg/mL ; 7.24e-07 mol/L
	Class	Poorly soluble
Pharmacokinetics	GI Absorption	High
	BBB Permeation	No
	P-gp Substrate	No
	Log K_p (Skin Permeation)	−4.55 cm/s
Druglikeness	Lipinski	Yes; 0 violation
	Ghose	No; 1 violation: WLOGP > 5.6
	Veber	Yes
	Egan	No; 1 violation: WLOGP > 5.88
	Muegge	No; 1 violation: XLOGP3 > 5
	Bioavailability Score	0.55
Medicinal Chemistry	PAINS	0 alert
	Brenk	1 alert: isolated_alkene
	Leadlikeness	No; 2 violations: MW>350, XLOGP3>3.5
	Synthetic accessibility	4.41

Abbreviations: SMILES, Simplified Molecular Input Line Entry System; Num., number; arom., aromatic; Csp3, fraction of sp^3-hybridized carbons; H-bond, hydrogen bond; TPSA, topological polar surface area; Log $P_{o/w}$, octanol–water partition coefficient; XLOGP3, XlogP3 lipophilicity model; WLOGP, Wildman–Crippen lipophilicity model; MLOGP, Moriguchi lipophilicity model; SILICOS-IT, SILICOS-IT lipophilicity/solubility model; Log S, logarithm of aqueous solubility; ESOL, estimated solubility model (Delaney); Ali, Ali solubility model; GI, gastrointestinal; BBB, blood–brain barrier; P-gp, P-glycoprotein; Log *K*p, logarithm of skin permeation coefficient; PAINS, pan-assay interference compounds.
